# Oncogenic Role of *miR-15a-3p* in 13q Amplicon-Driven Colorectal Adenoma-to-Carcinoma Progression

**DOI:** 10.1371/journal.pone.0132495

**Published:** 2015-07-06

**Authors:** Florence L. M. de Groen, Lisette M. Timmer, Renee X. Menezes, Begona Diosdado, Erik Hooijberg, Gerrit A. Meijer, Renske D. M. Steenbergen, Beatriz Carvalho

**Affiliations:** 1 Department of Pathology, VU University Medical Center, Amsterdam, the Netherlands; 2 Department of Epidemiology and Biostatistics, VU University Medical Center, Amsterdam, the Netherlands; 3 Department of Pathology, The Netherlands Cancer Institute, Amsterdam, the Netherlands; The University of Hong Kong, CHINA

## Abstract

Progression from colorectal adenoma to carcinoma is strongly associated with an accumulation of genomic alterations, including gain of chromosome 13. This gain affects the whole q arm and is present in 40%–60% of all colorectal cancers (CRCs). Several genes located at this amplicon are known to be overexpressed in carcinomas due to copy number dosage. A subset of these genes, including the *mir-17~92* cluster, are functionally involved in CRC development. The present study set out to explore whether apart from *mir-17~92*, other miRNAs located at the 13q amplicon show a copy number dependent dosage effect that may contribute to 13q-driven colorectal adenoma-to-carcinoma progression. Integration of publically available miRNA expression, target mRNA expression and DNA copy number data from 125 CRCs yielded three miRNAs, *miR-15a*, *-17*, and *-20a*, of which high expression levels were significantly correlated with a 13q gain and which influenced target mRNA expression. These results could be confirmed by qRT-PCR in a series of 100 colon adenomas and carcinomas.Functional analysis of both mature miRNAs encoded by *mir-15a*, i.e. *miR-15a-5p* and *miR-15a-3p*, showed that silencing of *miR-15a-3p* significantly inhibited viability of CRC cells. Integration of *miR-15a* expression levels with mRNA expression data of predicted target genes identified *mitochondrial uncoupling protein 2* (*UCP2)* and *COP9 signalosome subunit 2 (COPS2) *as candidates with significantly decreased expression in CRCs with 13q gain. Upon silencing of *miR-15a-3p*, mRNA expression of both genes increased in CRC cells, supporting *miR-15a-3p* mediated regulation of *UPC2* and *COPS2* expression. In conclusion, significant overexpression of *miR-15a-3p* due to gain of 13q is functionally relevant in CRC, with *UCP2* and *COPS2* as candidate target genes. Taken together our findings suggest that *miR-15a-3p* may contribute to adenoma-to-carcinoma progression.

## Introduction

The development of colorectal cancer (CRC) is marked by the accumulation of several recurrent chromosomal alterations, including gains of 8q, 13q, and 20q and losses of 8p, 15q, 17p and 18q [1–3], which can lead to altered expression of oncogenes and tumour suppressor genes [4,5]. In fact, for a number of genes identified to be differentially expressed in a copy number dependent manner, functional relevance has been demonstrated. For instance, *DIS3*, *LNX2* and *CDK8* at 13q were recently described to play a role in CRC development due to 13q gain-dependent overexpression [6–8]. The same holds true for *AURKA* and *TPX2* located at 20q, which were found to promote 20q amplicon-driven colorectal adenoma to carcinoma progression [9].

In addition to protein-encoding genes, DNA copy number changes may also affect expression of microRNAs (miRNAs) [10,11]. MiRNAs are a family of small non-coding RNA molecules that play an important role in the regulation of many cellular processes by targeting the 3’ UTR of mRNA molecules, thereby leading to gene silencing [12]. Dysregulation of miRNA expression has been shown to play an important role in several human diseases, including cancer [13].

In CRC, altered expression of a number of miRNAs, due to chromosomal alterations or other mechanisms like epigenetic modifications, has been described. A well-documented example is the altered expression of the oncogenic miRNA cluster, *mir-17~92* [14]. We previously showed that increased *mir-17~92* expression was linked to copy number gain of 13q and increased *c-MYC* expression during colorectal adenoma to carcinoma progression [15]. Moreover, upregulation of *mir-17~92* by c-*MYC* showed pro-angiogenic activity in colonocytes [16].

Chromosome 13 is gained in 40–60% of CRCs and is strongly associated with colorectal adenoma to carcinoma progression [1,2,5]. This gain mostly encompasses the entire q-arm of chromosome 13 [6,17], raising the question whether next to coding genes and the *mir-17~92* cluster, expression of other miRNAs located at this region is also affected by copy number dosage and may contribute to colorectal adenoma to carcinoma progression.

The present study aimed to identify additional candidate oncomiRs located at 13q in CRC. To this end, as an unbiased approach, we analysed all miRNAs mapping at 13q. Following validation of DNA copy number-dependent overexpression of 13q miRNAs by integrative analysis of DNA copy number and expression data-sets, the functional role of selected miRNAs was investigated in CRC cell lines using loss-of-function assays. Candidate target genes were identified by integration of miRNA expression levels with mRNA expression levels.

## Materials and Methods

### TCGA data

Data of 125 CRC samples from The Cancer Genome Atlas (TCGA) on DNA-copy number, mRNA and miRNA expression were downloaded from the TCGA Data Portal (https://tcga-data.nci.nih.gov/tcga/tcgaDataType.jsp). Additional information of all the cases used in this study (sample ID, platform, data level and file name) can be found in S1 Table. In all cases level 2 data (i.e. normalised signals per probe or probe set) were obtained, except for the SNP data, for which only level 3 was available (i.e. segmented DNA copy number data). The latter were re-formatted to represent the copy number measurements on 30,000 equally spaced locations on the genome. More detailed information on the data types downloaded can be found at the TCGA Data Portal.

### Integration of expression and copy number data

**Fig 1:**
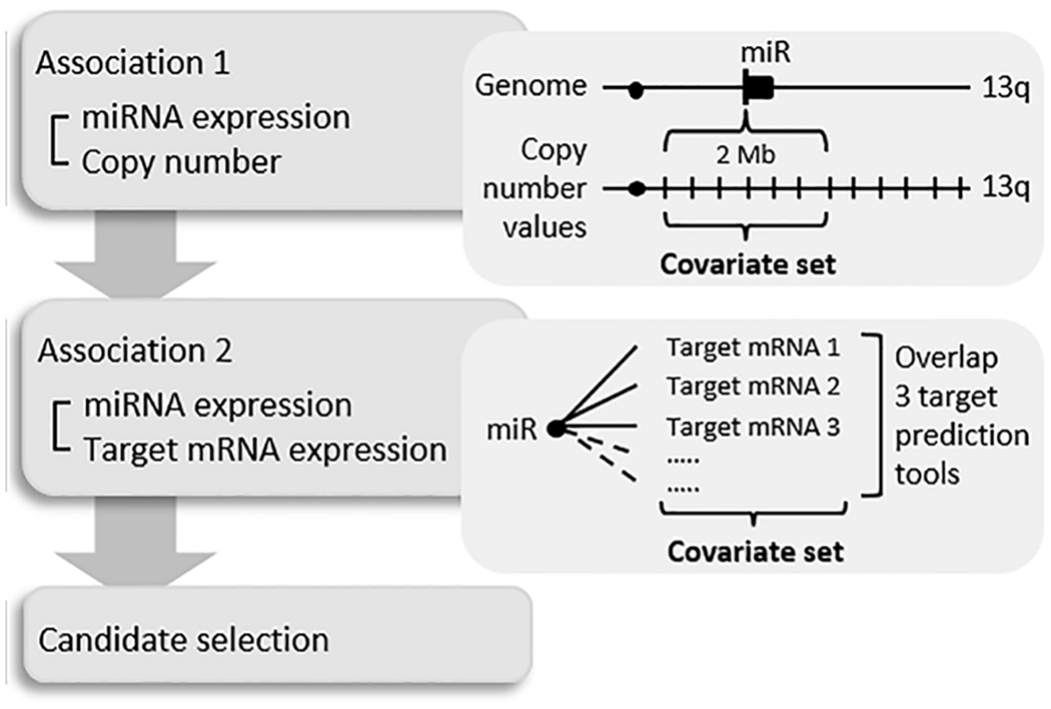
Schematic overview of in silico analysis pipeline used in this study. First level (association 1) shows a graphical representation of integration analysis of miRNA expression with copy number (CN) data. CN values for miRNAs on 13q were determined by combining values within a 2 Mb window surrounding the start of the miRNA. These values defined the covariate set used to determine association with miRNA expression. Second level (association 2) gives a graphical representation of the association of miRNA expression with predicted target mRNA expression. For each miRNA on 13q target mRNAs were determined by combining three or more target prediction tools. Expression levels of these target mRNAs defined the covariate set used to determine association with miRNA expression.

To investigate 13q miRNAs of which expression was regulated by DNA copy number changes, associations between DNA copy number and miRNA expression levels were studied. To this end we used an integrated approach, based on the use of covariate sets rather than single covariates, to determine subtle consistent associations with copy number alterations of a chromosomal region. The covariate set was defined by considering each miRNA probe individually and all copy number measurements within a 2Mb-window around the genomic start position of the miRNA. The analysis pipeline and definition of the covariate set is graphically presented in Fig 1 (association 1). The method used is implemented in the BioConductor package SIM [18]. This procedure finds miRNAs of which expression is likely to be regulated by DNA copy number changes within this 2MB-window, by assigning one p-value to each miRNA. Multiple testing correction was done using the false-discovery rate (FDR) step-down procedure of Benjamini & Hoghberg [19]. This analysis yielded a list of FDR values for miRNAs, of which expression is likely to be regulated by copy number changes in these samples. MiRNAs with FDR<0.05 were considered significant.

### Target mRNA and miRNA expression integration

To identify which miRNAs are more likely to be functional, we studied their association with expression of predicted target mRNAs in the 125 CRC TCGA samples. To this end we used the approach developed by van Iterson et al [20]. In short, for each miRNA, results of 4 target-prediction tools, namely TargetScan, PITA, microCosm and Mirtarbase [21–24], were combined to yield a list of possible mRNA targets. For further (i.e. second) analysis, candidate mRNA targets that were predicted by three or more prediction tools were selected. This list of target mRNAs defined the covariate set for this second analysis (Fig 1, association 2). Subsequently, a global test was used [25] to test for associations between miRNA and mRNA expression, yielding one p-value per miRNA. Here the same statistical test was used as for the miRNA-copy number analysis, but now using the mRNA target list as covariate set. These p-values were corrected for multiple testing using the Benjamini & Hochberg FDR [19]. FDR values <0.05 were considered significant. For each significant miRNA, we subsequently prioritised the mRNA targets using the p-value derived from an additional global test applied to the miRNA and each individual mRNA target. We selected for further analyses mRNA targets that had an individual p-value of <0.01.

In order to analyse expression patterns of several selected target mRNAs, previously generated expression data were used of a panel of colorectal adenomas (n = 37) and carcinomas (n = 31), available at GEO under accession number GSE8067 [5]. Expression levels in both groups were compared using the Mann-Whitney U-test, considering p-values <0.05 as significant.

### Tumour samples

A series of 100 snap-frozen colorectal tumour samples (48 adenomas and 52 carcinomas) prospectively collected at the VU University Medical Center in Amsterdam was used in this study. RNA from these samples was isolated using TRIzol (Invitrogen, Life technologies, Bleiswijk, The Netherlands), following the supplier’s instructions. For a subset of these samples (12 adenomas and 15 carcinomas) whole genome DNA copy number status was known (5). For the remaining samples copy number status of chromosome 13q was determined by multiplex ligation-dependent probe amplification (MLPA) as described previously [26].

### Quantitative reverse transcription-PCR

MiRNA expression levels in colon adenomas and carcinomas of *miR-15a-5p*, *miR-15a-3p*, *miR-17* and *miR-20a* were analysed using TaqMan MicroRNA Assays (assays: 000389, 002419, 002308, 000580 respectively) according to the manufacturer’s instructions (Applied Biosystems, Life Technologies). Relative miRNA expression levels were determined using the 2(-ΔΔCt)-method [27]. Data were normalised using *miR-16* as a reference gene (Applied Biosystems, Life technologies).

In cell lines miRNA expression levels of *miR-15a-3p*, *miR-15a-5p* and *miR-17* were analysed using the miRCURY LNA Universal RT microRNA PCR (Exiqon, Vedbaek, Denmark) following the manufacturer’s instructions. PCRs were run on an ABI 7500 Fast Real-time PCR system (Life technologies). Relative miRNA expression levels were determined using the 2(-ΔΔCt)-method [27]. Data were normalised using the small nucleolar RNA transcript RNU43 as a reference gene (Exiqon).

Messenger RNA (mRNA) expression levels of predicted targets (i.e. *UCP2* and *COPS2*) were analysed following cDNA synthesis using a mix of oligo(dT) and random hexamers (iScript cDNA Synthesis Kit; Bio-Rad, Veenendaal, The Netherlands). PCR reactions were performed using SYBR Green PCR mastermix (Life Technologies) with 0.5 μM of each primer (*UCP2*: 5’-CACCGTGAGACCTTACAAAGCC-3’, 3’-TGCTACGTCCCAGGAGATGG-5’; *COPS2*: 5’-CGCCAGTTACATCAGTCGTGC-3’, 3’-AGTGGATGAGGGATGGCAGAC-5’) and 20 ng of cDNA. Amplification reactions were performed for 50 cycles with an annealing temperature of 60°C, using a ViiA7 PCR system (Life Technologies). Data were normalised using β-2-microglobulin as a reference gene [28] and relative mRNA expression levels were determined using the 2(-ΔΔCt)-method [27]. Expression levels of miRNAs and target genes were compared using Student’s t-test, considering p-values <0.05 as significant.

### Cell culture and transfections

The CRC cell line SW480 was kindly provided Dr. G.J. Peters, Department of Oncology, VU University Medical Center. SW480 was cultured in Dulbecco’s modified Eagle’s medium (Lonza, Wijchen, The Netherlands) supplemented with 10% fetal bovine serum (PAA, Cölbe, Germany), 2 mM L-glutamine (Life Technologies), 100 IU/ml sodium-penicillin and 100 μg/ml streptomycin. Cultures were maintained at 37°C in a humidified atmosphere of 5% CO_2_.

SW480 was transiently transfected with 40 nM of mirCURY LNA microRNA power inhibitor directed against *miR-15a-3p*, *miR-15a-5p* and *miR-17* (Assays: 426841–00, 426842–00, 426848–00; Exiqon). *Negative control A* was used as non-targeting control (Assays: 199020–04 and 199020–00; Exiqon). Cells were seeded in 6-wells cell-culture plates and transfections were performed after 18 to 24 hours. Lipofectamine 2000 Reagent (Life technologies) was used as transfection reagent following manufacturer’s instructions.

### Cell viability

Cell viability was measured using the 3-(4,5-dimethylthiazolyl-2)-2,5-diphenyl tetrazolium bromide assay (MTT; MP Biomedicals, Eindhoven, The Netherlands). Twenty-four hours post-transfection with the antagomirs, cells were seeded in 96-wells culture plates for the MTT assay. Simultaneously, RNA was extracted from a subset of the cells to determine the miRNA expression levels. MTT measurements were performed at t = 0 and subsequently after 3 and 6 days. The relative viability was determined by subtracting the measurement at day 0 from subsequent time points. All measurements were performed in triplicate (technical replicates) and at least 2 independent experiments were performed (biological replicates). Relative viability of cells was compared using Student’s *t*-test, with p-values <0.05 considered significant.

## Results

### MiR-15a shows copy number-associated differential expression in CRC

In the present study, publically available TCGA data from 125 CRC samples were used to analyse both miRNA expression levels and DNA copy number levels of the loci involved on chromosome 13 [17]. According to miRBase release 21 (www.mirbase.org), there are 40 miRNA located at chromosome 13 [29]. For 14 out of these 40 miRNAs on 13q, expression data were available, and 6 of these were significantly overexpressed in CRCs due to copy number gain of 13q, i.e. *miR-15a*, *-17*, *-19a*, *-20a*, *19b-1* and *-92a-1* (Table 1; FDR<0.05). These miRNAs all belong to two well-known clusters of miRNAs on chromosome 13, i.e. *mir-15a/16* and *mir-17~92*.

**Table 1:** Association of miRNA expression with 13q copy number status and target mRNA expression.

miRNA	Chr	Start (bp position)	Copy number (FDR)	Target mRNA exp (FDR)
hsa-mir-20a	13q	90801327	0.000	0.000
hsa-mir-92a-1	13q	90801618	0.000	n.d.
hsa-mir-17	13q	90800874	0.000	0.001
hsa-mir-15a	13q	49521268	0.000	0.010
hsa-mir-19a	13q	90801194	0.001	0.052
hsa-mir-19b-1	13q	90801463	0.002	n.d.
hsa-mir-16-1	13q	49521123	0.160	n.d.
hsa-mir-18a	13q	90801011	0.255	0.017
hsa-mir-320d-1	13q	40199964	0.360	n.d.
hsa-mir-622	13q	89681504	0.428	0.633
hsa-mir-623	13q	98806372	0.779	0.050
hsa-mir-1297	13q	53784118	0.807	0.984
hsa-mir-1267	13q	106981565	0.964	0.989
hsa-mir-621	13q	40282935	0.969	0.906

MiRNAs are ordered by significance of FDR of miRNA expression versus copy number. Chr, Chromosome; bp, base pair; exp, expression; n.d., no data; Significance considered at FDR<0.05.

Next to miRNA expression data, mRNA expression data were also available for the same panel of 125 CRCs. Using these data we examined whether overexpression of these miRNAs on 13q resulted in changes in the expression of predicted target genes [20]. This analysis showed that, for *miR-15a*, *-17* and *-20a*, overexpression due to copy number also significantly influenced predicted target mRNA expression (Table 1; FDR<0.05).

**Fig 2:**
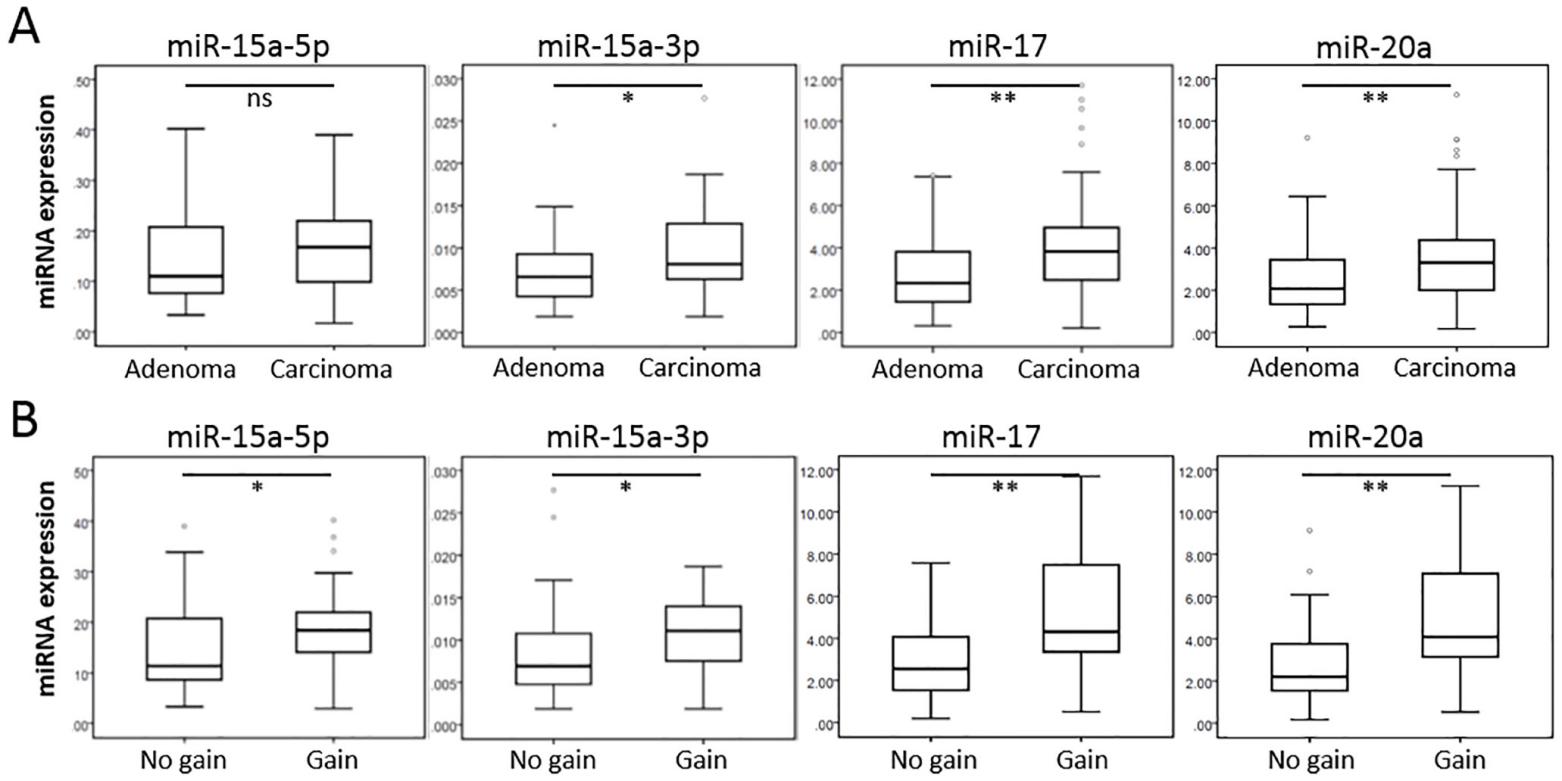
Analysis of miRNA expression in colorectal adenomas and carcinomas. A) Box plots comparing relative expression determined by qRT-PCR in adenomas (n = 48) versus carcinomas (n = 52) of four top candidate miRNAs, *miR-15a-3p*, *miR-15a-5p*, *miR-17* and *miR-20a*. B) Box plots comparing miRNA expression between tumours (adenomas and carcinomas) with (n = 24) or without (n = 73) 13q gain. P-values, determined by Mann-Whitney U-test, ≤ 0.05 are considered significant (* p ≤ 0.05; ** p < 0.01).

Next we analysed by qRT-PCR the expression levels of the top candidate miRNAs found in the *in silico* analyses in a series of 100 colon adenoma and carcinoma samples. Given the fact that *mir-15a* encodes for two mature miRNA strands, *miR-15a-5p* and *miR-15a-3p*, both were studied using strand-specific qRT-PCR. In line with the *in silico* data, expression levels of *miR-15a-3p*, *miR-17* and *miR-20a*, were significantly higher in carcinomas compared to adenomas (Fig 2A). However, expression of *miR-15a-5p* was not significantly increased. Comparison of tumours with 13q gain with tumours without 13q gain showed that expression levels of all four miRNAs were significantly higher in tumours with gain (Fig 2B), corroborating the results from the *in silico* analysis.

### MiR-15a-3p inhibits viability of CRC cells

Based on these analyses, *miR-15a*, *-17* and *-20a* were identified as candidate oncogenic miRNAs involved in CRC. Of these, both *miR-17* and *-20a* have recently been described as oncogenes in CRC and prostate cancer [30–32], confirming the validity of the approach. MiR-15a on the other hand has been described to function as a tumour suppressor in chronic lymphocytic leukaemia (CLL) [33,34] and, more recently, in CRC [35]. To further support our current observations pointing to an oncogenic role of *miR-15a* in CRC, we set out to determine the functional relevance of *miR-15a* overexpression in CRC. Both strands of *miR-15a*, *miR-15a-5p* and *miR-15a-3p*, were studied using strand-specific miRNA inhibitors (antagomirs). An antagomir directed against *miR-17*, silencing of which was shown to decrease viability of CRC cells, was included as a positive control [30]. A non-targeting antagomir was included as a negative control.

**Fig 3:**
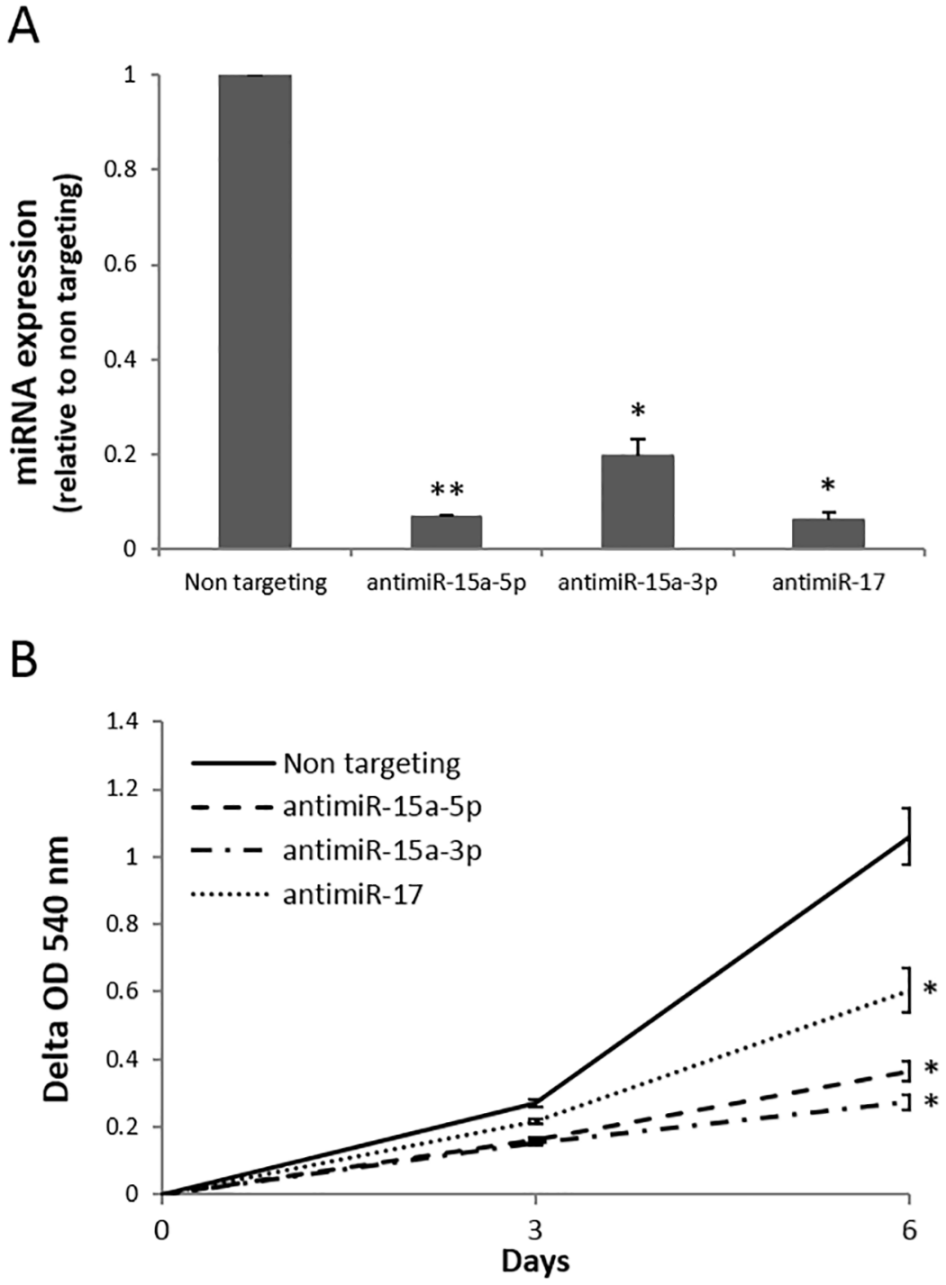
Functional analysis of the effect of *miR-15a-5p*, *-3p* and *miR-17* silencing in CRC cell line SW480. A) Representative example of expression levels of miRNAs upon silencing using antagomirs compared to a non-targeting control (**p<0.01; *p<0.05). B) Cell viability determined by MTT assay upon miRNA silencing in SW480 compared to non-targeting control (* p<0.05).

The antagomirs were introduced into the SW480 CRC cell line, which has a gain of 13q encompassing the *miR-15a* locus. Efficiency of silencing varied per experiment between 75%-97%, 60–80% and 94%-96% for *miR-15a-5p*, *miR-15a-3p* and *miR-17*, respectively (Fig 3A). Silencing of *miR-15a-3p* and *miR-17* led to a significant decrease in cell viability (Fig 3B). Silencing of *miR-15a-5p* also decreased cell viability significantly, although results were less consistent throughout replicate experiments (data not shown).

### *UCP2* and *COPS2* as potential targets of *miR-15a-3p* in CRC

By integrating miRNA expression profiles with mRNA expression profiles of predicted targets in 125 CRCs, we identified candidate target genes of *miR-15a* [20]. The top five are shown in Table 2 (p <0.01). Of these five candidate target genes, four genes (*TYRO3*, *UCP2*, *COPS2*, *RASGEF1B*) were negatively associated with *miR-15a* expression, whereas (*RNF125*) was positively associated.

**Table 2:** Significantly affected predicted target genes of miR-15a.

Gene symbol	Chr	p-value	Association
TYRO3	15q	0.001	-
UCP2	11q	0.001	-
RNF125	18q	0.001	+
RASGEF1B	4q	0.008	-
COPS2	15q	0.010	-

Genes are ordered by significance (NCBI build 37). Significance at p-value <0.01. Positive and negative association denotes association of mRNA target expression versus expression of miR-15a. Chr, chromosome.

**Fig 4:**
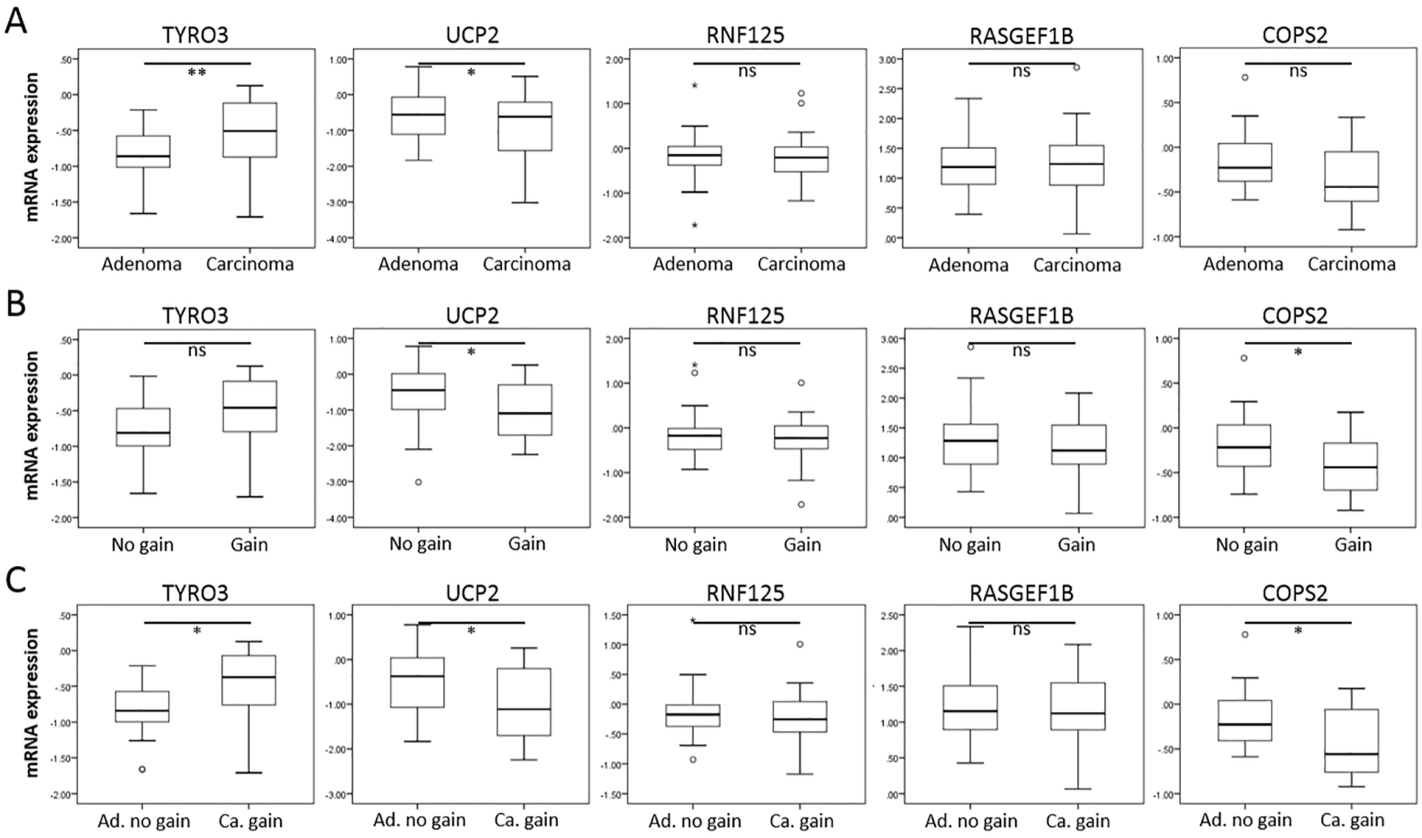
Analysis of target gene expression in colorectal adenomas and carcinomas. A) Box plots comparing relative mRNA expression (determined by expression microarray) in adenomas (n = 37) versus carcinomas (n = 31) of 5 target genes, *TYRO3*, *UCP2*, *RNF125*, *RASGEF1B* and *COPS2*. B) Box plots comparing mRNA expression between tumours (adenomas and carcinomas) with (n = 18) or without (n = 44) 13q gain. C) Box plots comparing mRNA expression in adenomas without 13q gain (n = 30) to carcinomas with 13q gain (n = 14). P-values, determined by Mann-Whitney U-test, ≤ 0.05 are considered significant (* p ≤ 0.05; ** p < 0.01; ns, not significant).

Since copy number gain of chromosome 13 has been associated with progression from colorectal adenoma-to-carcinoma, and current data show that this gain is associated with overexpression of *miR-15a*, we next evaluated whether the expression levels of the top five predicted target genes would show a decrease in carcinomas compared to adenomas, in conjunction with 13q gain. To this end, previously obtained array-CGH and mRNA expression microarray data of a panel of 37 colon adenomas and 31 carcinomas were used [5]. In this set, expression of *UCP2* was significantly decreased in carcinomas compared to adenomas (Fig 4A). Both *UCP2* and *COPS2* were significantly decreased in adenomas and carcinomas with a 13q gain compared to those without (Fig 4B). The comparison of adenomas without 13q gain to carcinomas with 13q gain, showed for both *UCP2* and *COPS2* significantly decreased expression in carcinomas with 13q gain (Fig 4C). In the case of *TYRO3*, a significant increase in expression was observed in both carcinomas (compared to adenomas), and in samples with 13q gain (compared to tumours without 13q gain). *RNF125* and *RASGEF1B* expression levels were not related to progression or 13q copy number gain.

**Fig 5:**
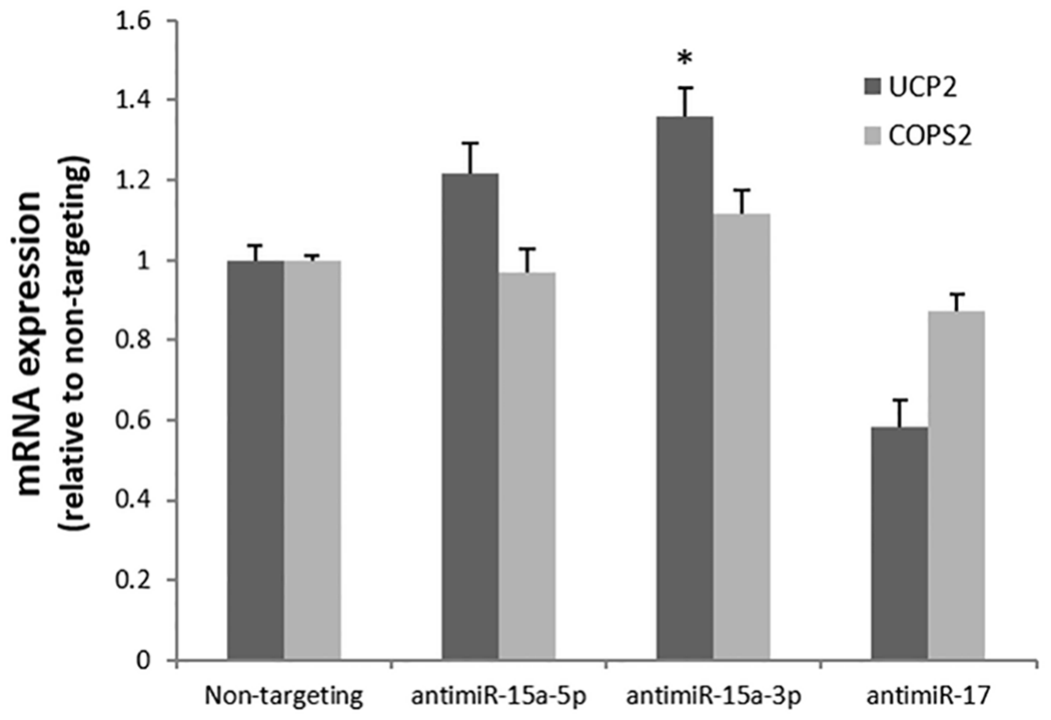
Analysis of mRNA expression levels of target genes in colorectal cancer cell line SW480. Relative expression of *UCP2* and *COPS2* in SW480 cells silenced for *miR-15a-5p*, *-3p* and *miR-17* compared to a non-targeting control (* p<0.05).

To further establish a role of *miR-15a* overexpression in *UCP2 and COPS2* downregulation in CRC, mRNA expression levels of both genes were determined in SW480 cells, in which either *miR15a-3p*, *miR15a-5p* or control *miR-17* were silenced. Compared to the non-targeting control, mRNA expression levels of *UCP2* and, to a lesser extent, *COPS2* were increased upon silencing of *miR-15a-3p* (Fig 5). Hardly any effect was seen when using antagomirs against *miR-15a-5p* or miR-17 (Fig 5). These results indicate that *miR-15a-3p* indeed targets both *UCP2* and *COPS2* in SW480 CRC cells.

## Discussion

Here we investigated miRNAs that are overexpressed in CRC due to copy number gain of chromosome 13q. Analysis of publicly available TCGA miRNA expression and DNA copy number data of 125 CRC samples [17] revealed that *miR-15a*, *-17* and *-20a* were significantly upregulated in CRC in association with copy number gain of 13q, and that *miR-15a*, *-17* and *-20a* significantly influenced expression of target genes. For both *miR-20a* and -*17*, an oncogenic role in CRC has been established [30,36]. Interestingly, for *miR-15a* actually a tumour suppressor role has been claimed [35]. The present results however indicate that *miR-15a*, in particular *miR-15a-3p*, functions as an oncogenic miRNA in CRC. Silencing of *miR-15a-3p* significantly decreased viability of CRC cells.

*MiR-15a* is part of the *mir-15a/16-1* cluster located at chromosome 13q14, which is encoded by its host gene *deleted in leukaemia 2* (*DLEU2*) [34,37]. To date, members of the *mir-15a/16-1* cluster have been described as tumour suppressor miRNAs in several types of cancer [34,35,38–40]. In chronic lymphocytic leukaemia, inactivation of these miRNAs occurs through a chromosomal loss at the locus of the *mir-15a/16-1* cluster [34]. In multiple myeloma, expression of *mir-15a/16-1* was decreased whereas expression of its target gene *VEGF-A* was increased. Moreover, in these tumours expression of *mir-15a/16-1* was shown to inhibit tumour formation by targeting pro-angiogenic factor *VEGF-A* [39]. Similarly, in prostate cancer the expression of the miRNA cluster was downregulated, which was associated with upregulation of target genes *BCL2*, *CCND1* and *WNT3A*. Knockdown of *miR-15a* and *miR-16* in an *in vitro* model enhanced cancer progression by affecting cell survival, proliferation and invasion [38].

In CRC, *miR-16* of this cluster had already been described to have a function as tumour suppressor [41,42]. More recently however, the entire *mir-15a/16-1* cluster was investigated and found to target *AP4* in CRC, downregulation of which resulted in induction of mesenchymal-epithelial transition (MET), inhibition of migration and invasion, and induction of cell cycle arrest [35]. Additionally, expression of *DLEU2*, the gene encoding the *mir-15a/16-1* cluster, was repressed in CRC samples compared to normal colon tissue and inversely correlated to *AP4* expression. Altogether, those data suggest that *miR-15a* expression is decreased in CRC compared to normal tissue, which seems contradictory to the findings in the current study. In fact, the TCGA data presented here, also show that in CRC samples the expression of *miR-15a* actually increases in association with a DNA copy number gain of chromosome 13q. This gain is a common event in CRC, and in particular, in the progression from colorectal adenoma to carcinoma [2,6,17].

With respect to the role of the *mir-15a/16-1* cluster in CRC, it is important to realize that the present study specifically focused on the association with a 13q gain involved in adenoma to carcinoma progression, whereas the above mentioned study of Shi *et al*. focused on CRC metastasis, representing a later event in the carcinogenic process [35]. More importantly, we specifically identified *miR-15a-3p* as the driver strand and did not find evidence for the whole cluster to be involved. Recent research also showed that *miR-15a-3p* plays a tumour suppressor role in several cancer cell lines by targeting *BCL-X*_*L*_. However, no significant effect was seen in CRC cell lines [40]. It is known that both the 5p and 3p strands of a miRNA can be expressed in cells and regulate target gene expression [43,44]. The expression of either strand can also be tissue-specific [45]. We therefore speculate that expression of different mature miRNAs from the *mir-15a/16-1* cluster may have different roles in CRC, perhaps depending on the stage of CRC.

Evaluation of *miR-15a-3p* expression in a series of 48 adenomas and 52 carcinomas, for which 13q copy number status was known, showed that *miR-15a-3p* was significantly higher expressed in carcinomas compared to adenomas in a DNA copy number-dependent manner. These results corroborate an oncogenic role for increased *miR-15a-3p* expression on the 13q amplicon.

By combining the results of target prediction algorithms with mRNA expression profile data of CRCs, *UCP2* and *COPS2* were identified as candidate target genes of *miR-15a*. In support of this observation, upregulation of *UCP2* and, to a lesser extent, of *COPS2* was found in CRC cells when *miR-15a-3p* was silenced, compared to control transfectants. The absence of a similar effect in *miR-15a-5p* silenced CRC cells is consistent with our functional data showing that both strands seem to act independently. The present data indicate that *UCP2*, and potentially also *COPS2*, may function as tumour suppressors in CRC. Interestingly, overexpression of *UCP2* in human pancreatic cancer and glioblastoma cell lines was recently described to repress malignancy by controlling mitochondrial function and redirecting energy production away from glycolysis [46]. Also *COPS2* was described as a putative tumour suppressor gene in a panel of different cancer types, where loss-of-function showed partial bypass of cellular senescence [47]. Altogether, one could hypothesize that tumour cells would benefit from the inhibition of these genes through miRNA-mediated modulation.

According to the most recent release of the miRNA database (miRBase release 21), there are 40 miRNAs located at chromosome 13q. However, for this study we only had expression data of 14 13q-mapping miRNAs available, as expression levels of the remaining miRNAs were undetectable or not measured. Therefore, it cannot be excluded that additional miRNAs located at 13q may also be involved in CRC.

In summary, *miR-15a-3p* is gained and overexpressed in CRC, and this overexpression affects cell viability, supporting an oncogenic role of this miRNA in CRC. As a consequence, *miR-15a-3p* may play a role in the 13q gain-driven colorectal adenoma-to-carcinoma progression.

## Supporting Information

S1 Table Overview of all the TCGA cases used in this study. Additional information on the data of 125 cases used from the TCGA. For all cases the sample ID, type of data (platform type), platform used, data level and the corresponding file names are listed. More information on the data used can be found at the TCGA Data Portal (https://tcga-data.nci.nih.gov/tcga/tcgaDataType.jsp).
(DOC)
