# Supplementary material for: Oncogenic Role of miR-15a-3p in 13q Amplicon-Driven Colorectal Adenoma-to-Carcinoma Progression
Source: PLoS One. 2015 Jul 6;10(7):e0132495. doi: 10.1371/journal.pone.0132495 (PMC4492490; doi:10.1371/journal.pone.0132495)
Supplement: S1 Table — Additional information on the data of 125 cases used from the TCGA. For all cases the sample ID, type of data (platform type), platform used, data level and the corresponding file names are listed. More information on the data used can be found at the TCGA Data Portal (https://tcga-data.nci.nih.gov/tcga/tcgaDataType.jsp). (DOC) [file pone.0132495.s001.doc]

**Supplementary table 1: Overview of data of 125 TCGA cases used in this study.**

| **Sample ID** | **Platform type** | **Platform** | **Data level** | **File name** |
| --- | --- | --- | --- | --- |
| TCGA-A6-2670-01 | Expression-Genes | AgilentG4502A_07_3 | 2 | US82800149_251976011004_S01_GE2_105_Dec08.txt_lmean.out.logratio.probe.tcga_level2.data.txt |
| TCGA-A6-2672-01 | Expression-Genes | AgilentG4502A_07_3 | 2 | US82800149_251976011362_S01_GE2_105_Dec08.txt_lmean.out.logratio.probe.tcga_level2.data.txt |
| TCGA-A6-2674-01 | Expression-Genes | AgilentG4502A_07_3 | 2 | US82800149_251976011000_S01_GE2_105_Dec08.txt_lmean.out.logratio.probe.tcga_level2.data.txt |
| TCGA-A6-2676-01 | Expression-Genes | AgilentG4502A_07_3 | 2 | US82800149_251976011365_S01_GE2_105_Dec08.txt_lmean.out.logratio.probe.tcga_level2.data.txt |
| TCGA-A6-2677-01 | Expression-Genes | AgilentG4502A_07_3 | 2 | US82800149_251976011008_S01_GE2_105_Dec08.txt_lmean.out.logratio.probe.tcga_level2.data.txt |
| TCGA-A6-2678-01 | Expression-Genes | AgilentG4502A_07_3 | 2 | US82800149_251976011014_S01_GE2_105_Dec08.txt_lmean.out.logratio.probe.tcga_level2.data.txt |
| TCGA-A6-2683-01 | Expression-Genes | AgilentG4502A_07_3 | 2 | US82800149_251976011005_S01_GE2_105_Dec08.txt_lmean.out.logratio.probe.tcga_level2.data.txt |
| TCGA-A6-3807-01 | Expression-Genes | AgilentG4502A_07_3 | 2 | US82800149_251976011762_S01_GE2_105_Dec08.txt_lmean.out.logratio.probe.tcga_level2.data.txt |
| TCGA-A6-3810-01 | Expression-Genes | AgilentG4502A_07_3 | 2 | US82800149_251976011774_S01_GE2_105_Dec08.txt_lmean.out.logratio.probe.tcga_level2.data.txt |
| TCGA-AA-3514-01 | Expression-Genes | AgilentG4502A_07_3 | 2 | US82800149_251976011024_S01_GE2_105_Dec08.txt_lmean.out.logratio.probe.tcga_level2.data.txt |
| TCGA-AA-3516-01 | Expression-Genes | AgilentG4502A_07_3 | 2 | US82800149_251976011368_S01_GE2_105_Dec08.txt_lmean.out.logratio.probe.tcga_level2.data.txt |
| TCGA-AA-3517-01 | Expression-Genes | AgilentG4502A_07_3 | 2 | US82800149_251976011030_S01_GE2_105_Dec08.txt_lmean.out.logratio.probe.tcga_level2.data.txt |
| TCGA-AA-3518-01 | Expression-Genes | AgilentG4502A_07_3 | 2 | US82800149_251976011371_S01_GE2_105_Dec08.txt_lmean.out.logratio.probe.tcga_level2.data.txt |
| TCGA-AA-3519-01 | Expression-Genes | AgilentG4502A_07_3 | 2 | US82800149_251976011342_S01_GE2_105_Dec08.txt_lmean.out.logratio.probe.tcga_level2.data.txt |
| TCGA-AA-3521-01 | Expression-Genes | AgilentG4502A_07_3 | 2 | US82800149_251976011001_S01_GE2_105_Dec08.txt_lmean.out.logratio.probe.tcga_level2.data.txt |
| TCGA-AA-3522-01 | Expression-Genes | AgilentG4502A_07_3 | 2 | US82800149_251976011009_S01_GE2_105_Dec08.txt_lmean.out.logratio.probe.tcga_level2.data.txt |
| TCGA-AA-3524-01 | Expression-Genes | AgilentG4502A_07_3 | 2 | US82800149_251976011015_S01_GE2_105_Dec08.txt_lmean.out.logratio.probe.tcga_level2.data.txt |
| TCGA-AA-3526-01 | Expression-Genes | AgilentG4502A_07_3 | 2 | US82800149_251976011006_S01_GE2_105_Dec08.txt_lmean.out.logratio.probe.tcga_level2.data.txt |
| TCGA-AA-3527-01 | Expression-Genes | AgilentG4502A_07_3 | 2 | US82800149_251976011025_S01_GE2_105_Dec08.txt_lmean.out.logratio.probe.tcga_level2.data.txt |
| TCGA-AA-3529-01 | Expression-Genes | AgilentG4502A_07_3 | 2 | US82800149_251976011031_S01_GE2_105_Dec08.txt_lmean.out.logratio.probe.tcga_level2.data.txt |
| TCGA-AA-3530-01 | Expression-Genes | AgilentG4502A_07_3 | 2 | US82800149_251976011781_S01_GE2_105_Dec08.txt_lmean.out.logratio.probe.tcga_level2.data.txt |
| TCGA-AA-3531-01 | Expression-Genes | AgilentG4502A_07_3 | 2 | US82800149_251976011343_S01_GE2_105_Dec08.txt_lmean.out.logratio.probe.tcga_level2.data.txt |
| TCGA-AA-3532-01 | Expression-Genes | AgilentG4502A_07_3 | 2 | US82800149_251976010996_S01_GE2_105_Dec08.txt_lmean.out.logratio.probe.tcga_level2.data.txt |
| TCGA-AA-3534-01 | Expression-Genes | AgilentG4502A_07_3 | 2 | US82800149_251976011002_S01_GE2_105_Dec08.txt_lmean.out.logratio.probe.tcga_level2.data.txt |
| TCGA-AA-3538-01 | Expression-Genes | AgilentG4502A_07_3 | 2 | US82800149_251976011010_S01_GE2_105_Dec08.txt_lmean.out.logratio.probe.tcga_level2.data.txt |
| TCGA-AA-3542-01 | Expression-Genes | AgilentG4502A_07_3 | 2 | US82800149_251976011016_S01_GE2_105_Dec08.txt_lmean.out.logratio.probe.tcga_level2.data.txt |
| TCGA-AA-3543-01 | Expression-Genes | AgilentG4502A_07_3 | 2 | US82800149_251976011258_S01_GE2_105_Dec08.txt_lmean.out.logratio.probe.tcga_level2.data.txt |
| TCGA-AA-3544-01 | Expression-Genes | AgilentG4502A_07_3 | 2 | US82800149_251976011020_S01_GE2_105_Dec08.txt_lmean.out.logratio.probe.tcga_level2.data.txt |
| TCGA-AA-3548-01 | Expression-Genes | AgilentG4502A_07_3 | 2 | US82800149_251976011026_S01_GE2_105_Dec08.txt_lmean.out.logratio.probe.tcga_level2.data.txt |
| TCGA-AA-3549-01 | Expression-Genes | AgilentG4502A_07_3 | 2 | US82800149_251976011032_S01_GE2_105_Dec08.txt_lmean.out.logratio.probe.tcga_level2.data.txt |
| TCGA-AA-3552-01 | Expression-Genes | AgilentG4502A_07_3 | 2 | US82800149_251976011345_S01_GE2_105_Dec08.txt_lmean.out.logratio.probe.tcga_level2.data.txt |
| TCGA-AA-3553-01 | Expression-Genes | AgilentG4502A_07_3 | 2 | US82800149_251976010997_S01_GE2_105_Dec08.txt_lmean.out.logratio.probe.tcga_level2.data.txt |
| TCGA-AA-3554-01 | Expression-Genes | AgilentG4502A_07_3 | 2 | US82800149_251976011418_S01_GE2_105_Dec08.txt_lmean.out.logratio.probe.tcga_level2.data.txt |
| TCGA-AA-3555-01 | Expression-Genes | AgilentG4502A_07_3 | 2 | US82800149_251976011003_S01_GE2_105_Dec08.txt_lmean.out.logratio.probe.tcga_level2.data.txt |
| TCGA-AA-3556-01 | Expression-Genes | AgilentG4502A_07_3 | 2 | US82800149_251976011011_S01_GE2_105_Dec08.txt_lmean.out.logratio.probe.tcga_level2.data.txt |
| TCGA-AA-3558-01 | Expression-Genes | AgilentG4502A_07_3 | 2 | US82800149_251976011017_S01_GE2_105_Dec08.txt_lmean.out.logratio.probe.tcga_level2.data.txt |
| TCGA-AA-3560-01 | Expression-Genes | AgilentG4502A_07_3 | 2 | US82800149_251976011021_S01_GE2_105_Dec08.txt_lmean.out.logratio.probe.tcga_level2.data.txt |
| TCGA-AA-3561-01 | Expression-Genes | AgilentG4502A_07_3 | 2 | US82800149_251976011027_S01_GE2_105_Dec08.txt_lmean.out.logratio.probe.tcga_level2.data.txt |
| TCGA-AA-3562-01 | Expression-Genes | AgilentG4502A_07_3 | 2 | US82800149_251976011033_S01_GE2_105_Dec08.txt_lmean.out.logratio.probe.tcga_level2.data.txt |
| TCGA-AA-3664-01 | Expression-Genes | AgilentG4502A_07_3 | 2 | US82800149_251976011479_S01_GE2_105_Dec08.txt_lmean.out.logratio.probe.tcga_level2.data.txt |
| TCGA-AA-3666-01 | Expression-Genes | AgilentG4502A_07_3 | 2 | US82800149_251976011485_S01_GE2_105_Dec08.txt_lmean.out.logratio.probe.tcga_level2.data.txt |
| TCGA-AA-3667-01 | Expression-Genes | AgilentG4502A_07_3 | 2 | US82800149_251976011629_S01_GE2_105_Dec08.txt_lmean.out.logratio.probe.tcga_level2.data.txt |
| TCGA-AA-3672-01 | Expression-Genes | AgilentG4502A_07_3 | 2 | US82800149_251976011634_S01_GE2_105_Dec08.txt_lmean.out.logratio.probe.tcga_level2.data.txt |
| TCGA-AA-3673-01 | Expression-Genes | AgilentG4502A_07_3 | 2 | US82800149_251976011639_S01_GE2_105_Dec08.txt_lmean.out.logratio.probe.tcga_level2.data.txt |
| TCGA-AA-3678-01 | Expression-Genes | AgilentG4502A_07_3 | 2 | US82800149_251976011649_S01_GE2_105_Dec08.txt_lmean.out.logratio.probe.tcga_level2.data.txt |
| TCGA-AA-3679-01 | Expression-Genes | AgilentG4502A_07_3 | 2 | US82800149_251976011654_S01_GE2_105_Dec08.txt_lmean.out.logratio.probe.tcga_level2.data.txt |
| TCGA-AA-3680-01 | Expression-Genes | AgilentG4502A_07_3 | 2 | US82800149_251976011480_S01_GE2_105_Dec08.txt_lmean.out.logratio.probe.tcga_level2.data.txt |
| TCGA-AA-3681-01 | Expression-Genes | AgilentG4502A_07_3 | 2 | US82800149_251976011486_S01_GE2_105_Dec08.txt_lmean.out.logratio.probe.tcga_level2.data.txt |
| TCGA-AA-3684-01 | Expression-Genes | AgilentG4502A_07_3 | 2 | US82800149_251976011630_S01_GE2_105_Dec08.txt_lmean.out.logratio.probe.tcga_level2.data.txt |
| TCGA-AA-3685-01 | Expression-Genes | AgilentG4502A_07_3 | 2 | US82800149_251976011635_S01_GE2_105_Dec08.txt_lmean.out.logratio.probe.tcga_level2.data.txt |
| TCGA-AA-3688-01 | Expression-Genes | AgilentG4502A_07_3 | 2 | US82800149_251976011640_S01_GE2_105_Dec08.txt_lmean.out.logratio.probe.tcga_level2.data.txt |
| TCGA-AA-3692-01 | Expression-Genes | AgilentG4502A_07_3 | 2 | US82800149_251976011645_S01_GE2_105_Dec08.txt_lmean.out.logratio.probe.tcga_level2.data.txt |
| TCGA-AA-3693-01 | Expression-Genes | AgilentG4502A_07_3 | 2 | US82800149_251976011650_S01_GE2_105_Dec08.txt_lmean.out.logratio.probe.tcga_level2.data.txt |
| TCGA-AA-3695-01 | Expression-Genes | AgilentG4502A_07_3 | 2 | US82800149_251976011655_S01_GE2_105_Dec08.txt_lmean.out.logratio.probe.tcga_level2.data.txt |
| TCGA-AA-3696-01 | Expression-Genes | AgilentG4502A_07_3 | 2 | US82800149_251976011481_S01_GE2_105_Dec08.txt_lmean.out.logratio.probe.tcga_level2.data.txt |
| TCGA-AA-3710-01 | Expression-Genes | AgilentG4502A_07_3 | 2 | US82800149_251976011787_S01_GE2_105_Dec08.txt_lmean.out.logratio.probe.tcga_level2.data.txt |
| TCGA-AA-3715-01 | Expression-Genes | AgilentG4502A_07_3 | 2 | US82800149_251976011487_S01_GE2_105_Dec08.txt_lmean.out.logratio.probe.tcga_level2.data.txt |
| TCGA-AA-3811-01 | Expression-Genes | AgilentG4502A_07_3 | 2 | US82800149_251976011793_S01_GE2_105_Dec08.txt_lmean.out.logratio.probe.tcga_level2.data.txt |
| TCGA-AA-3812-01 | Expression-Genes | AgilentG4502A_07_3 | 2 | US82800149_251976011631_S01_GE2_105_Dec08.txt_lmean.out.logratio.probe.tcga_level2.data.txt |
| TCGA-AA-3814-01 | Expression-Genes | AgilentG4502A_07_3 | 2 | US82800149_251976011636_S01_GE2_105_Dec08.txt_lmean.out.logratio.probe.tcga_level2.data.txt |
| TCGA-AA-3818-01 | Expression-Genes | AgilentG4502A_07_3 | 2 | US82800149_251976011641_S01_GE2_105_Dec08.txt_lmean.out.logratio.probe.tcga_level2.data.txt |
| TCGA-AA-3819-01 | Expression-Genes | AgilentG4502A_07_3 | 2 | US82800149_251976011646_S01_GE2_105_Dec08.txt_lmean.out.logratio.probe.tcga_level2.data.txt |
| TCGA-AA-3821-01 | Expression-Genes | AgilentG4502A_07_3 | 2 | US82800149_251976011763_S01_GE2_105_Dec08.txt_lmean.out.logratio.probe.tcga_level2.data.txt |
| TCGA-AA-3831-01 | Expression-Genes | AgilentG4502A_07_3 | 2 | US82800149_251976011651_S01_GE2_105_Dec08.txt_lmean.out.logratio.probe.tcga_level2.data.txt |
| TCGA-AA-3833-01 | Expression-Genes | AgilentG4502A_07_3 | 2 | US82800149_251976011656_S01_GE2_105_Dec08.txt_lmean.out.logratio.probe.tcga_level2.data.txt |
| TCGA-AA-3837-01 | Expression-Genes | AgilentG4502A_07_3 | 2 | US82800149_251976011482_S01_GE2_105_Dec08.txt_lmean.out.logratio.probe.tcga_level2.data.txt |
| TCGA-AA-3842-01 | Expression-Genes | AgilentG4502A_07_3 | 2 | US82800149_251976011942_S01_GE2_105_Dec08.txt_lmean.out.logratio.probe.tcga_level2.data.txt |
| TCGA-AA-3844-01 | Expression-Genes | AgilentG4502A_07_3 | 2 | US82800149_251976011948_S01_GE2_105_Dec08.txt_lmean.out.logratio.probe.tcga_level2.data.txt |
| TCGA-AA-3845-01 | Expression-Genes | AgilentG4502A_07_3 | 2 | US82800149_251976011776_S01_GE2_105_Dec08.txt_lmean.out.logratio.probe.tcga_level2.data.txt |
| TCGA-AA-3846-01 | Expression-Genes | AgilentG4502A_07_3 | 2 | US82800149_251976011782_S01_GE2_105_Dec08.txt_lmean.out.logratio.probe.tcga_level2.data.txt |
| TCGA-AA-3848-01 | Expression-Genes | AgilentG4502A_07_3 | 2 | US82800149_251976011632_S01_GE2_105_Dec08.txt_lmean.out.logratio.probe.tcga_level2.data.txt |
| TCGA-AA-3850-01 | Expression-Genes | AgilentG4502A_07_3 | 2 | US82800149_251976011788_S01_GE2_105_Dec08.txt_lmean.out.logratio.probe.tcga_level2.data.txt |
| TCGA-AA-3851-01 | Expression-Genes | AgilentG4502A_07_3 | 2 | US82800149_251976011794_S01_GE2_105_Dec08.txt_lmean.out.logratio.probe.tcga_level2.data.txt |
| TCGA-AA-3852-01 | Expression-Genes | AgilentG4502A_07_3 | 2 | US82800149_251976011637_S01_GE2_105_Dec08.txt_lmean.out.logratio.probe.tcga_level2.data.txt |
| TCGA-AA-3854-01 | Expression-Genes | AgilentG4502A_07_3 | 2 | US82800149_251976011642_S01_GE2_105_Dec08.txt_lmean.out.logratio.probe.tcga_level2.data.txt |
| TCGA-AA-3855-01 | Expression-Genes | AgilentG4502A_07_3 | 2 | US82800149_251976011800_S01_GE2_105_Dec08.txt_lmean.out.logratio.probe.tcga_level2.data.txt |
| TCGA-AA-3856-01 | Expression-Genes | AgilentG4502A_07_3 | 2 | US82800149_251976011647_S01_GE2_105_Dec08.txt_lmean.out.logratio.probe.tcga_level2.data.txt |
| TCGA-AA-3858-01 | Expression-Genes | AgilentG4502A_07_3 | 2 | US82800149_251976011652_S01_GE2_105_Dec08.txt_lmean.out.logratio.probe.tcga_level2.data.txt |
| TCGA-AA-3860-01 | Expression-Genes | AgilentG4502A_07_3 | 2 | US82800149_251976011657_S01_GE2_105_Dec08.txt_lmean.out.logratio.probe.tcga_level2.data.txt |
| TCGA-AA-3864-01 | Expression-Genes | AgilentG4502A_07_3 | 2 | US82800149_251976011770_S01_GE2_105_Dec08.txt_lmean.out.logratio.probe.tcga_level2.data.txt |
| TCGA-AA-3866-01 | Expression-Genes | AgilentG4502A_07_3 | 2 | US82800149_251976011777_S01_GE2_105_Dec08.txt_lmean.out.logratio.probe.tcga_level2.data.txt |
| TCGA-AA-3867-01 | Expression-Genes | AgilentG4502A_07_3 | 2 | US82800149_251976011783_S01_GE2_105_Dec08.txt_lmean.out.logratio.probe.tcga_level2.data.txt |
| TCGA-AA-3869-01 | Expression-Genes | AgilentG4502A_07_3 | 2 | US82800149_251976011789_S01_GE2_105_Dec08.txt_lmean.out.logratio.probe.tcga_level2.data.txt |
| TCGA-AA-3870-01 | Expression-Genes | AgilentG4502A_07_3 | 2 | US82800149_251976011795_S01_GE2_105_Dec08.txt_lmean.out.logratio.probe.tcga_level2.data.txt |
| TCGA-AA-3872-01 | Expression-Genes | AgilentG4502A_07_3 | 2 | US82800149_251976011801_S01_GE2_105_Dec08.txt_lmean.out.logratio.probe.tcga_level2.data.txt |
| TCGA-AA-3875-01 | Expression-Genes | AgilentG4502A_07_3 | 2 | US82800149_251976011483_S01_GE2_105_Dec08.txt_lmean.out.logratio.probe.tcga_level2.data.txt |
| TCGA-AA-3877-01 | Expression-Genes | AgilentG4502A_07_3 | 2 | US82800149_251976011765_S01_GE2_105_Dec08.txt_lmean.out.logratio.probe.tcga_level2.data.txt |
| TCGA-AA-3930-01 | Expression-Genes | AgilentG4502A_07_3 | 2 | US82800149_251976011944_S01_GE2_105_Dec08.txt_lmean.out.logratio.probe.tcga_level2.data.txt |
| TCGA-AA-3939-01 | Expression-Genes | AgilentG4502A_07_3 | 2 | US82800149_251976011771_S01_GE2_105_Dec08.txt_lmean.out.logratio.probe.tcga_level2.data.txt |
| TCGA-AA-3941-01 | Expression-Genes | AgilentG4502A_07_3 | 2 | US82800149_251976011778_S01_GE2_105_Dec08.txt_lmean.out.logratio.probe.tcga_level2.data.txt |
| TCGA-AA-3947-01 | Expression-Genes | AgilentG4502A_07_3 | 2 | US82800149_251976011784_S01_GE2_105_Dec08.txt_lmean.out.logratio.probe.tcga_level2.data.txt |
| TCGA-AA-3949-01 | Expression-Genes | AgilentG4502A_07_3 | 2 | US82800149_251976011790_S01_GE2_105_Dec08.txt_lmean.out.logratio.probe.tcga_level2.data.txt |
| TCGA-AA-3950-01 | Expression-Genes | AgilentG4502A_07_3 | 2 | US82800149_251976011796_S01_GE2_105_Dec08.txt_lmean.out.logratio.probe.tcga_level2.data.txt |
| TCGA-AA-3952-01 | Expression-Genes | AgilentG4502A_07_3 | 2 | US82800149_251976011802_S01_GE2_105_Dec08.txt_lmean.out.logratio.probe.tcga_level2.data.txt |
| TCGA-AA-3955-01 | Expression-Genes | AgilentG4502A_07_3 | 2 | US82800149_251976011766_S01_GE2_105_Dec08.txt_lmean.out.logratio.probe.tcga_level2.data.txt |
| TCGA-AA-3956-01 | Expression-Genes | AgilentG4502A_07_3 | 2 | US82800149_251976011945_S01_GE2_105_Dec08.txt_lmean.out.logratio.probe.tcga_level2.data.txt |
| TCGA-AA-3966-01 | Expression-Genes | AgilentG4502A_07_3 | 2 | US82800149_251976012135_S01_GE2_105_Dec08.txt_lmean.out.logratio.probe.tcga_level2.data.txt |
| TCGA-AA-3968-01 | Expression-Genes | AgilentG4502A_07_3 | 2 | US82800149_251976011772_S01_GE2_105_Dec08.txt_lmean.out.logratio.probe.tcga_level2.data.txt |
| TCGA-AA-3971-01 | Expression-Genes | AgilentG4502A_07_3 | 2 | US82800149_251976011785_S01_GE2_105_Dec08.txt_lmean.out.logratio.probe.tcga_level2.data.txt |
| TCGA-AA-3972-01 | Expression-Genes | AgilentG4502A_07_3 | 2 | US82800149_251976011791_S01_GE2_105_Dec08.txt_lmean.out.logratio.probe.tcga_level2.data.txt |
| TCGA-AA-3973-01 | Expression-Genes | AgilentG4502A_07_3 | 2 | US82800149_251976011797_S01_GE2_105_Dec08.txt_lmean.out.logratio.probe.tcga_level2.data.txt |
| TCGA-AA-3975-01 | Expression-Genes | AgilentG4502A_07_3 | 2 | US82800149_251976011803_S01_GE2_105_Dec08.txt_lmean.out.logratio.probe.tcga_level2.data.txt |
| TCGA-AA-3976-01 | Expression-Genes | AgilentG4502A_07_3 | 2 | US82800149_251976011767_S01_GE2_105_Dec08.txt_lmean.out.logratio.probe.tcga_level2.data.txt |
| TCGA-AA-3977-01 | Expression-Genes | AgilentG4502A_07_3 | 2 | US82800149_251976011946_S01_GE2_105_Dec08.txt_lmean.out.logratio.probe.tcga_level2.data.txt |
| TCGA-AA-3979-01 | Expression-Genes | AgilentG4502A_07_3 | 2 | US82800149_251976011773_S01_GE2_105_Dec08.txt_lmean.out.logratio.probe.tcga_level2.data.txt |
| TCGA-AA-3982-01 | Expression-Genes | AgilentG4502A_07_3 | 2 | US82800149_251976011786_S01_GE2_105_Dec08.txt_lmean.out.logratio.probe.tcga_level2.data.txt |
| TCGA-AA-3984-01 | Expression-Genes | AgilentG4502A_07_3 | 2 | US82800149_251976011792_S01_GE2_105_Dec08.txt_lmean.out.logratio.probe.tcga_level2.data.txt |
| TCGA-AA-3986-01 | Expression-Genes | AgilentG4502A_07_3 | 2 | US82800149_251976011798_S01_GE2_105_Dec08.txt_lmean.out.logratio.probe.tcga_level2.data.txt |
| TCGA-AA-3989-01 | Expression-Genes | AgilentG4502A_07_3 | 2 | US82800149_251976011804_S01_GE2_105_Dec08.txt_lmean.out.logratio.probe.tcga_level2.data.txt |
| TCGA-AA-3994-01 | Expression-Genes | AgilentG4502A_07_3 | 2 | US82800149_251976012137_S01_GE2_105_Dec08.txt_lmean.out.logratio.probe.tcga_level2.data.txt |
| TCGA-AA-A00L-01 | Expression-Genes | AgilentG4502A_07_3 | 2 | US82800149_251976011389_S01_GE2_105_Dec08.txt_lmean.out.logratio.probe.tcga_level2.data.txt |
| TCGA-AA-A00O-01 | Expression-Genes | AgilentG4502A_07_3 | 2 | US82800149_251976011452_S01_GE2_105_Dec08.txt_lmean.out.logratio.probe.tcga_level2.data.txt |
| TCGA-AA-A00Q-01 | Expression-Genes | AgilentG4502A_07_3 | 2 | US82800149_251976011393_S01_GE2_105_Dec08.txt_lmean.out.logratio.probe.tcga_level2.data.txt |
| TCGA-AA-A00W-01 | Expression-Genes | AgilentG4502A_07_3 | 2 | US82800149_251976011114_S01_GE2_105_Dec08.txt_lmean.out.logratio.probe.tcga_level2.data.txt |
| TCGA-AA-A010-01 | Expression-Genes | AgilentG4502A_07_3 | 2 | US82800149_251976011455_S01_GE2_105_Dec08.txt_lmean.out.logratio.probe.tcga_level2.data.txt |
| TCGA-AA-A01D-01 | Expression-Genes | AgilentG4502A_07_3 | 2 | US82800149_251976011940_S01_GE2_105_Dec08.txt_lmean.out.logratio.probe.tcga_level2.data.txt |
| TCGA-AA-A024-01 | Expression-Genes | AgilentG4502A_07_3 | 2 | US82800149_251976011459_S01_GE2_105_Dec08.txt_lmean.out.logratio.probe.tcga_level2.data.txt |
| TCGA-AA-A029-01 | Expression-Genes | AgilentG4502A_07_3 | 2 | US82800149_251976011441_S01_GE2_105_Dec08.txt_lmean.out.logratio.probe.tcga_level2.data.txt |
| TCGA-AA-A02E-01 | Expression-Genes | AgilentG4502A_07_3 | 2 | US82800149_251976011445_S01_GE2_105_Dec08.txt_lmean.out.logratio.probe.tcga_level2.data.txt |
| TCGA-AA-A02F-01 | Expression-Genes | AgilentG4502A_07_3 | 2 | US82800149_251976011450_S01_GE2_105_Dec08.txt_lmean.out.logratio.probe.tcga_level2.data.txt |
| TCGA-AA-A02H-01 | Expression-Genes | AgilentG4502A_07_3 | 2 | US82800149_251976011457_S01_GE2_105_Dec08.txt_lmean.out.logratio.probe.tcga_level2.data.txt |
| TCGA-AA-A02J-01 | Expression-Genes | AgilentG4502A_07_3 | 2 | US82800149_251976011837_S01_GE2_105_Dec08.txt_lmean.out.logratio.probe.tcga_level2.data.txt |
| TCGA-AA-A02R-01 | Expression-Genes | AgilentG4502A_07_3 | 2 | US82800149_251976011840_S01_GE2_105_Dec08.txt_lmean.out.logratio.probe.tcga_level2.data.txt |
| TCGA-AY-4070-01 | Expression-Genes | AgilentG4502A_07_3 | 2 | US82800149_251976012138_S01_GE2_105_Dec08.txt_lmean.out.logratio.probe.tcga_level2.data.txt |
| TCGA-AY-4071-01 | Expression-Genes | AgilentG4502A_07_3 | 2 | US82800149_251976012139_S01_GE2_105_Dec08.txt_lmean.out.logratio.probe.tcga_level2.data.txt |
| TCGA-A6-2670-01 | miRNASeq | IlluminaGA_miRNASeq | 3 | bcgsc.ca__IlluminaGA_miRNASeq__TCGA-A6-2670-01A-02T-0822-13__mirna_quantification.txt |
| TCGA-A6-2672-01 | miRNASeq | IlluminaGA_miRNASeq | 3 | bcgsc.ca__IlluminaGA_miRNASeq__TCGA-A6-2672-01A-01T-0827-13__mirna_quantification.txt |
| TCGA-A6-2674-01 | miRNASeq | IlluminaGA_miRNASeq | 3 | bcgsc.ca__IlluminaGA_miRNASeq__TCGA-A6-2674-01A-02T-0822-13__mirna_quantification.txt |
| TCGA-A6-2676-01 | miRNASeq | IlluminaGA_miRNASeq | 3 | bcgsc.ca__IlluminaGA_miRNASeq__TCGA-A6-2676-01A-01T-0827-13__mirna_quantification.txt |
| TCGA-A6-2677-01 | miRNASeq | IlluminaGA_miRNASeq | 3 | bcgsc.ca__IlluminaGA_miRNASeq__TCGA-A6-2677-01A-01T-0822-13__mirna_quantification.txt |
| TCGA-A6-2678-01 | miRNASeq | IlluminaGA_miRNASeq | 3 | bcgsc.ca__IlluminaGA_miRNASeq__TCGA-A6-2678-01A-01T-0822-13__mirna_quantification.txt |
| TCGA-A6-2683-01 | miRNASeq | IlluminaGA_miRNASeq | 3 | bcgsc.ca__IlluminaGA_miRNASeq__TCGA-A6-2683-01A-01T-0822-13__mirna_quantification.txt |
| TCGA-A6-3807-01 | miRNASeq | IlluminaGA_miRNASeq | 3 | bcgsc.ca__IlluminaGA_miRNASeq__TCGA-A6-3807-01A-01T-1021-13__mirna_quantification.txt |
| TCGA-A6-3810-01 | miRNASeq | IlluminaGA_miRNASeq | 3 | bcgsc.ca__IlluminaGA_miRNASeq__TCGA-A6-3810-01A-01T-1021-13__mirna_quantification.txt |
| TCGA-AA-3514-01 | miRNASeq | IlluminaGA_miRNASeq | 3 | bcgsc.ca__IlluminaGA_miRNASeq__TCGA-AA-3514-01A-02T-0822-13__mirna_quantification.txt |
| TCGA-AA-3516-01 | miRNASeq | IlluminaGA_miRNASeq | 3 | bcgsc.ca__IlluminaGA_miRNASeq__TCGA-AA-3516-01A-02T-0827-13__mirna_quantification.txt |
| TCGA-AA-3517-01 | miRNASeq | IlluminaGA_miRNASeq | 3 | bcgsc.ca__IlluminaGA_miRNASeq__TCGA-AA-3517-01A-01T-0822-13__mirna_quantification.txt |
| TCGA-AA-3518-01 | miRNASeq | IlluminaGA_miRNASeq | 3 | bcgsc.ca__IlluminaGA_miRNASeq__TCGA-AA-3518-01A-02T-0827-13__mirna_quantification.txt |
| TCGA-AA-3519-01 | miRNASeq | IlluminaGA_miRNASeq | 3 | bcgsc.ca__IlluminaGA_miRNASeq__TCGA-AA-3519-01A-02T-0822-13__mirna_quantification.txt |
| TCGA-AA-3521-01 | miRNASeq | IlluminaGA_miRNASeq | 3 | bcgsc.ca__IlluminaGA_miRNASeq__TCGA-AA-3521-01A-01T-0822-13__mirna_quantification.txt |
| TCGA-AA-3522-01 | miRNASeq | IlluminaGA_miRNASeq | 3 | bcgsc.ca__IlluminaGA_miRNASeq__TCGA-AA-3522-01A-01T-0822-13__mirna_quantification.txt |
| TCGA-AA-3524-01 | miRNASeq | IlluminaGA_miRNASeq | 3 | bcgsc.ca__IlluminaGA_miRNASeq__TCGA-AA-3524-01A-02T-0822-13__mirna_quantification.txt |
| TCGA-AA-3526-01 | miRNASeq | IlluminaGA_miRNASeq | 3 | bcgsc.ca__IlluminaGA_miRNASeq__TCGA-AA-3526-01A-02T-0822-13__mirna_quantification.txt |
| TCGA-AA-3527-01 | miRNASeq | IlluminaGA_miRNASeq | 3 | bcgsc.ca__IlluminaGA_miRNASeq__TCGA-AA-3527-01A-01T-0822-13__mirna_quantification.txt |
| TCGA-AA-3529-01 | miRNASeq | IlluminaGA_miRNASeq | 3 | bcgsc.ca__IlluminaGA_miRNASeq__TCGA-AA-3529-01A-02T-0822-13__mirna_quantification.txt |
| TCGA-AA-3530-01 | miRNASeq | IlluminaGA_miRNASeq | 3 | bcgsc.ca__IlluminaGA_miRNASeq__TCGA-AA-3530-01A-01T-1021-13__mirna_quantification.txt |
| TCGA-AA-3531-01 | miRNASeq | IlluminaGA_miRNASeq | 3 | bcgsc.ca__IlluminaGA_miRNASeq__TCGA-AA-3531-01A-01T-0822-13__mirna_quantification.txt |
| TCGA-AA-3532-01 | miRNASeq | IlluminaGA_miRNASeq | 3 | bcgsc.ca__IlluminaGA_miRNASeq__TCGA-AA-3532-01A-01T-0822-13__mirna_quantification.txt |
| TCGA-AA-3534-01 | miRNASeq | IlluminaGA_miRNASeq | 3 | bcgsc.ca__IlluminaGA_miRNASeq__TCGA-AA-3534-01A-01T-0822-13__mirna_quantification.txt |
| TCGA-AA-3538-01 | miRNASeq | IlluminaGA_miRNASeq | 3 | bcgsc.ca__IlluminaGA_miRNASeq__TCGA-AA-3538-01A-01T-0822-13__mirna_quantification.txt |
| TCGA-AA-3542-01 | miRNASeq | IlluminaGA_miRNASeq | 3 | bcgsc.ca__IlluminaGA_miRNASeq__TCGA-AA-3542-01A-02T-0822-13__mirna_quantification.txt |
| TCGA-AA-3543-01 | miRNASeq | IlluminaGA_miRNASeq | 3 | bcgsc.ca__IlluminaGA_miRNASeq__TCGA-AA-3543-01A-01T-0827-13__mirna_quantification.txt |
| TCGA-AA-3544-01 | miRNASeq | IlluminaGA_miRNASeq | 3 | bcgsc.ca__IlluminaGA_miRNASeq__TCGA-AA-3544-01A-01T-0822-13__mirna_quantification.txt |
| TCGA-AA-3548-01 | miRNASeq | IlluminaGA_miRNASeq | 3 | bcgsc.ca__IlluminaGA_miRNASeq__TCGA-AA-3548-01A-01T-0822-13__mirna_quantification.txt |
| TCGA-AA-3549-01 | miRNASeq | IlluminaGA_miRNASeq | 3 | bcgsc.ca__IlluminaGA_miRNASeq__TCGA-AA-3549-01A-02T-0822-13__mirna_quantification.txt |
| TCGA-AA-3552-01 | miRNASeq | IlluminaGA_miRNASeq | 3 | bcgsc.ca__IlluminaGA_miRNASeq__TCGA-AA-3552-01A-01T-0822-13__mirna_quantification.txt |
| TCGA-AA-3553-01 | miRNASeq | IlluminaGA_miRNASeq | 3 | bcgsc.ca__IlluminaGA_miRNASeq__TCGA-AA-3553-01A-01T-0822-13__mirna_quantification.txt |
| TCGA-AA-3554-01 | miRNASeq | IlluminaGA_miRNASeq | 3 | bcgsc.ca__IlluminaGA_miRNASeq__TCGA-AA-3554-01A-01T-0827-13__mirna_quantification.txt |
| TCGA-AA-3555-01 | miRNASeq | IlluminaGA_miRNASeq | 3 | bcgsc.ca__IlluminaGA_miRNASeq__TCGA-AA-3555-01A-01T-0822-13__mirna_quantification.txt |
| TCGA-AA-3556-01 | miRNASeq | IlluminaGA_miRNASeq | 3 | bcgsc.ca__IlluminaGA_miRNASeq__TCGA-AA-3556-01A-01T-0822-13__mirna_quantification.txt |
| TCGA-AA-3558-01 | miRNASeq | IlluminaGA_miRNASeq | 3 | bcgsc.ca__IlluminaGA_miRNASeq__TCGA-AA-3558-01A-01T-0822-13__mirna_quantification.txt |
| TCGA-AA-3560-01 | miRNASeq | IlluminaGA_miRNASeq | 3 | bcgsc.ca__IlluminaGA_miRNASeq__TCGA-AA-3560-01A-01T-0822-13__mirna_quantification.txt |
| TCGA-AA-3561-01 | miRNASeq | IlluminaGA_miRNASeq | 3 | bcgsc.ca__IlluminaGA_miRNASeq__TCGA-AA-3561-01A-01T-0822-13__mirna_quantification.txt |
| TCGA-AA-3562-01 | miRNASeq | IlluminaGA_miRNASeq | 3 | bcgsc.ca__IlluminaGA_miRNASeq__TCGA-AA-3562-01A-02T-0822-13__mirna_quantification.txt |
| TCGA-AA-3664-01 | miRNASeq | IlluminaGA_miRNASeq | 3 | bcgsc.ca__IlluminaGA_miRNASeq__TCGA-AA-3664-01A-01T-0906-13__mirna_quantification.txt |
| TCGA-AA-3666-01 | miRNASeq | IlluminaGA_miRNASeq | 3 | bcgsc.ca__IlluminaGA_miRNASeq__TCGA-AA-3666-01A-02T-0906-13__mirna_quantification.txt |
| TCGA-AA-3667-01 | miRNASeq | IlluminaGA_miRNASeq | 3 | bcgsc.ca__IlluminaGA_miRNASeq__TCGA-AA-3667-01A-01T-0906-13__mirna_quantification.txt |
| TCGA-AA-3672-01 | miRNASeq | IlluminaGA_miRNASeq | 3 | bcgsc.ca__IlluminaGA_miRNASeq__TCGA-AA-3672-01A-01T-0906-13__mirna_quantification.txt |
| TCGA-AA-3673-01 | miRNASeq | IlluminaGA_miRNASeq | 3 | bcgsc.ca__IlluminaGA_miRNASeq__TCGA-AA-3673-01A-01T-0906-13__mirna_quantification.txt |
| TCGA-AA-3678-01 | miRNASeq | IlluminaGA_miRNASeq | 3 | bcgsc.ca__IlluminaGA_miRNASeq__TCGA-AA-3678-01A-01T-0906-13__mirna_quantification.txt |
| TCGA-AA-3679-01 | miRNASeq | IlluminaGA_miRNASeq | 3 | bcgsc.ca__IlluminaGA_miRNASeq__TCGA-AA-3679-01A-02T-0906-13__mirna_quantification.txt |
| TCGA-AA-3680-01 | miRNASeq | IlluminaGA_miRNASeq | 3 | bcgsc.ca__IlluminaGA_miRNASeq__TCGA-AA-3680-01A-01T-0906-13__mirna_quantification.txt |
| TCGA-AA-3681-01 | miRNASeq | IlluminaGA_miRNASeq | 3 | bcgsc.ca__IlluminaGA_miRNASeq__TCGA-AA-3681-01A-01T-0906-13__mirna_quantification.txt |
| TCGA-AA-3684-01 | miRNASeq | IlluminaGA_miRNASeq | 3 | bcgsc.ca__IlluminaGA_miRNASeq__TCGA-AA-3684-01A-02T-0906-13__mirna_quantification.txt |
| TCGA-AA-3685-01 | miRNASeq | IlluminaGA_miRNASeq | 3 | bcgsc.ca__IlluminaGA_miRNASeq__TCGA-AA-3685-01A-02T-0906-13__mirna_quantification.txt |
| TCGA-AA-3688-01 | miRNASeq | IlluminaGA_miRNASeq | 3 | bcgsc.ca__IlluminaGA_miRNASeq__TCGA-AA-3688-01A-01T-0906-13__mirna_quantification.txt |
| TCGA-AA-3692-01 | miRNASeq | IlluminaGA_miRNASeq | 3 | bcgsc.ca__IlluminaGA_miRNASeq__TCGA-AA-3692-01A-01T-0906-13__mirna_quantification.txt |
| TCGA-AA-3693-01 | miRNASeq | IlluminaGA_miRNASeq | 3 | bcgsc.ca__IlluminaGA_miRNASeq__TCGA-AA-3693-01A-01T-0906-13__mirna_quantification.txt |
| TCGA-AA-3695-01 | miRNASeq | IlluminaGA_miRNASeq | 3 | bcgsc.ca__IlluminaGA_miRNASeq__TCGA-AA-3695-01A-01T-0906-13__mirna_quantification.txt |
| TCGA-AA-3696-01 | miRNASeq | IlluminaGA_miRNASeq | 3 | bcgsc.ca__IlluminaGA_miRNASeq__TCGA-AA-3696-01A-01T-0906-13__mirna_quantification.txt |
| TCGA-AA-3710-01 | miRNASeq | IlluminaGA_miRNASeq | 3 | bcgsc.ca__IlluminaGA_miRNASeq__TCGA-AA-3710-01A-01T-1021-13__mirna_quantification.txt |
| TCGA-AA-3715-01 | miRNASeq | IlluminaGA_miRNASeq | 3 | bcgsc.ca__IlluminaGA_miRNASeq__TCGA-AA-3715-01A-01T-0906-13__mirna_quantification.txt |
| TCGA-AA-3811-01 | miRNASeq | IlluminaGA_miRNASeq | 3 | bcgsc.ca__IlluminaGA_miRNASeq__TCGA-AA-3811-01A-01T-1021-13__mirna_quantification.txt |
| TCGA-AA-3812-01 | miRNASeq | IlluminaGA_miRNASeq | 3 | bcgsc.ca__IlluminaGA_miRNASeq__TCGA-AA-3812-01A-01T-0906-13__mirna_quantification.txt |
| TCGA-AA-3814-01 | miRNASeq | IlluminaGA_miRNASeq | 3 | bcgsc.ca__IlluminaGA_miRNASeq__TCGA-AA-3814-01A-01T-0906-13__mirna_quantification.txt |
| TCGA-AA-3818-01 | miRNASeq | IlluminaGA_miRNASeq | 3 | bcgsc.ca__IlluminaGA_miRNASeq__TCGA-AA-3818-01A-01T-0906-13__mirna_quantification.txt |
| TCGA-AA-3819-01 | miRNASeq | IlluminaGA_miRNASeq | 3 | bcgsc.ca__IlluminaGA_miRNASeq__TCGA-AA-3819-01A-01T-0906-13__mirna_quantification.txt |
| TCGA-AA-3821-01 | miRNASeq | IlluminaGA_miRNASeq | 3 | bcgsc.ca__IlluminaGA_miRNASeq__TCGA-AA-3821-01A-01T-1021-13__mirna_quantification.txt |
| TCGA-AA-3831-01 | miRNASeq | IlluminaGA_miRNASeq | 3 | bcgsc.ca__IlluminaGA_miRNASeq__TCGA-AA-3831-01A-01T-0906-13__mirna_quantification.txt |
| TCGA-AA-3833-01 | miRNASeq | IlluminaGA_miRNASeq | 3 | bcgsc.ca__IlluminaGA_miRNASeq__TCGA-AA-3833-01A-01T-0906-13__mirna_quantification.txt |
| TCGA-AA-3837-01 | miRNASeq | IlluminaGA_miRNASeq | 3 | bcgsc.ca__IlluminaGA_miRNASeq__TCGA-AA-3837-01A-01T-0906-13__mirna_quantification.txt |
| TCGA-AA-3842-01 | miRNASeq | IlluminaGA_miRNASeq | 3 | bcgsc.ca__IlluminaGA_miRNASeq__TCGA-AA-3842-01A-01T-1021-13__mirna_quantification.txt |
| TCGA-AA-3844-01 | miRNASeq | IlluminaGA_miRNASeq | 3 | bcgsc.ca__IlluminaGA_miRNASeq__TCGA-AA-3844-01A-01T-1021-13__mirna_quantification.txt |
| TCGA-AA-3845-01 | miRNASeq | IlluminaGA_miRNASeq | 3 | bcgsc.ca__IlluminaGA_miRNASeq__TCGA-AA-3845-01A-01T-1021-13__mirna_quantification.txt |
| TCGA-AA-3846-01 | miRNASeq | IlluminaGA_miRNASeq | 3 | bcgsc.ca__IlluminaGA_miRNASeq__TCGA-AA-3846-01A-01T-1021-13__mirna_quantification.txt |
| TCGA-AA-3848-01 | miRNASeq | IlluminaGA_miRNASeq | 3 | bcgsc.ca__IlluminaGA_miRNASeq__TCGA-AA-3848-01A-01T-0906-13__mirna_quantification.txt |
| TCGA-AA-3850-01 | miRNASeq | IlluminaGA_miRNASeq | 3 | bcgsc.ca__IlluminaGA_miRNASeq__TCGA-AA-3850-01A-01T-1021-13__mirna_quantification.txt |
| TCGA-AA-3851-01 | miRNASeq | IlluminaGA_miRNASeq | 3 | bcgsc.ca__IlluminaGA_miRNASeq__TCGA-AA-3851-01A-01T-1021-13__mirna_quantification.txt |
| TCGA-AA-3852-01 | miRNASeq | IlluminaGA_miRNASeq | 3 | bcgsc.ca__IlluminaGA_miRNASeq__TCGA-AA-3852-01A-01T-0906-13__mirna_quantification.txt |
| TCGA-AA-3854-01 | miRNASeq | IlluminaGA_miRNASeq | 3 | bcgsc.ca__IlluminaGA_miRNASeq__TCGA-AA-3854-01A-01T-0906-13__mirna_quantification.txt |
| TCGA-AA-3855-01 | miRNASeq | IlluminaGA_miRNASeq | 3 | bcgsc.ca__IlluminaGA_miRNASeq__TCGA-AA-3855-01A-01T-1021-13__mirna_quantification.txt |
| TCGA-AA-3856-01 | miRNASeq | IlluminaGA_miRNASeq | 3 | bcgsc.ca__IlluminaGA_miRNASeq__TCGA-AA-3856-01A-01T-0906-13__mirna_quantification.txt |
| TCGA-AA-3858-01 | miRNASeq | IlluminaGA_miRNASeq | 3 | bcgsc.ca__IlluminaGA_miRNASeq__TCGA-AA-3858-01A-01T-0906-13__mirna_quantification.txt |
| TCGA-AA-3860-01 | miRNASeq | IlluminaGA_miRNASeq | 3 | bcgsc.ca__IlluminaGA_miRNASeq__TCGA-AA-3860-01A-02T-0906-13__mirna_quantification.txt |
| TCGA-AA-3864-01 | miRNASeq | IlluminaGA_miRNASeq | 3 | bcgsc.ca__IlluminaGA_miRNASeq__TCGA-AA-3864-01A-01T-1021-13__mirna_quantification.txt |
| TCGA-AA-3866-01 | miRNASeq | IlluminaGA_miRNASeq | 3 | bcgsc.ca__IlluminaGA_miRNASeq__TCGA-AA-3866-01A-01T-1021-13__mirna_quantification.txt |
| TCGA-AA-3867-01 | miRNASeq | IlluminaGA_miRNASeq | 3 | bcgsc.ca__IlluminaGA_miRNASeq__TCGA-AA-3867-01A-01T-1021-13__mirna_quantification.txt |
| TCGA-AA-3869-01 | miRNASeq | IlluminaGA_miRNASeq | 3 | bcgsc.ca__IlluminaGA_miRNASeq__TCGA-AA-3869-01A-01T-1021-13__mirna_quantification.txt |
| TCGA-AA-3870-01 | miRNASeq | IlluminaGA_miRNASeq | 3 | bcgsc.ca__IlluminaGA_miRNASeq__TCGA-AA-3870-01A-01T-1021-13__mirna_quantification.txt |
| TCGA-AA-3872-01 | miRNASeq | IlluminaGA_miRNASeq | 3 | bcgsc.ca__IlluminaGA_miRNASeq__TCGA-AA-3872-01A-01T-1021-13__mirna_quantification.txt |
| TCGA-AA-3875-01 | miRNASeq | IlluminaGA_miRNASeq | 3 | bcgsc.ca__IlluminaGA_miRNASeq__TCGA-AA-3875-01A-01T-0906-13__mirna_quantification.txt |
| TCGA-AA-3877-01 | miRNASeq | IlluminaGA_miRNASeq | 3 | bcgsc.ca__IlluminaGA_miRNASeq__TCGA-AA-3877-01A-01T-1021-13__mirna_quantification.txt |
| TCGA-AA-3930-01 | miRNASeq | IlluminaGA_miRNASeq | 3 | bcgsc.ca__IlluminaGA_miRNASeq__TCGA-AA-3930-01A-01T-1021-13__mirna_quantification.txt |
| TCGA-AA-3939-01 | miRNASeq | IlluminaGA_miRNASeq | 3 | bcgsc.ca__IlluminaGA_miRNASeq__TCGA-AA-3939-01A-01T-1021-13__mirna_quantification.txt |
| TCGA-AA-3941-01 | miRNASeq | IlluminaGA_miRNASeq | 3 | bcgsc.ca__IlluminaGA_miRNASeq__TCGA-AA-3941-01A-01T-1021-13__mirna_quantification.txt |
| TCGA-AA-3947-01 | miRNASeq | IlluminaGA_miRNASeq | 3 | bcgsc.ca__IlluminaGA_miRNASeq__TCGA-AA-3947-01A-01T-1021-13__mirna_quantification.txt |
| TCGA-AA-3949-01 | miRNASeq | IlluminaGA_miRNASeq | 3 | bcgsc.ca__IlluminaGA_miRNASeq__TCGA-AA-3949-01A-01T-1021-13__mirna_quantification.txt |
| TCGA-AA-3950-01 | miRNASeq | IlluminaGA_miRNASeq | 3 | bcgsc.ca__IlluminaGA_miRNASeq__TCGA-AA-3950-01A-02T-1021-13__mirna_quantification.txt |
| TCGA-AA-3952-01 | miRNASeq | IlluminaGA_miRNASeq | 3 | bcgsc.ca__IlluminaGA_miRNASeq__TCGA-AA-3952-01A-01T-1021-13__mirna_quantification.txt |
| TCGA-AA-3955-01 | miRNASeq | IlluminaGA_miRNASeq | 3 | bcgsc.ca__IlluminaGA_miRNASeq__TCGA-AA-3955-01A-02T-1021-13__mirna_quantification.txt |
| TCGA-AA-3956-01 | miRNASeq | IlluminaGA_miRNASeq | 3 | bcgsc.ca__IlluminaGA_miRNASeq__TCGA-AA-3956-01A-02T-1021-13__mirna_quantification.txt |
| TCGA-AA-3966-01 | miRNASeq | IlluminaGA_miRNASeq | 3 | bcgsc.ca__IlluminaGA_miRNASeq__TCGA-AA-3966-01A-01T-1114-13__mirna_quantification.txt |
| TCGA-AA-3968-01 | miRNASeq | IlluminaGA_miRNASeq | 3 | bcgsc.ca__IlluminaGA_miRNASeq__TCGA-AA-3968-01A-01T-1021-13__mirna_quantification.txt |
| TCGA-AA-3971-01 | miRNASeq | IlluminaGA_miRNASeq | 3 | bcgsc.ca__IlluminaGA_miRNASeq__TCGA-AA-3971-01A-01T-1021-13__mirna_quantification.txt |
| TCGA-AA-3972-01 | miRNASeq | IlluminaGA_miRNASeq | 3 | bcgsc.ca__IlluminaGA_miRNASeq__TCGA-AA-3972-01A-01T-1021-13__mirna_quantification.txt |
| TCGA-AA-3973-01 | miRNASeq | IlluminaGA_miRNASeq | 3 | bcgsc.ca__IlluminaGA_miRNASeq__TCGA-AA-3973-01A-01T-1021-13__mirna_quantification.txt |
| TCGA-AA-3975-01 | miRNASeq | IlluminaGA_miRNASeq | 3 | bcgsc.ca__IlluminaGA_miRNASeq__TCGA-AA-3975-01A-01T-1021-13__mirna_quantification.txt |
| TCGA-AA-3976-01 | miRNASeq | IlluminaGA_miRNASeq | 3 | bcgsc.ca__IlluminaGA_miRNASeq__TCGA-AA-3976-01A-01T-1021-13__mirna_quantification.txt |
| TCGA-AA-3977-01 | miRNASeq | IlluminaGA_miRNASeq | 3 | bcgsc.ca__IlluminaGA_miRNASeq__TCGA-AA-3977-01A-01T-1021-13__mirna_quantification.txt |
| TCGA-AA-3979-01 | miRNASeq | IlluminaGA_miRNASeq | 3 | bcgsc.ca__IlluminaGA_miRNASeq__TCGA-AA-3979-01A-01T-1021-13__mirna_quantification.txt |
| TCGA-AA-3982-01 | miRNASeq | IlluminaGA_miRNASeq | 3 | bcgsc.ca__IlluminaGA_miRNASeq__TCGA-AA-3982-01A-02T-1021-13__mirna_quantification.txt |
| TCGA-AA-3984-01 | miRNASeq | IlluminaGA_miRNASeq | 3 | bcgsc.ca__IlluminaGA_miRNASeq__TCGA-AA-3984-01A-02T-1021-13__mirna_quantification.txt |
| TCGA-AA-3986-01 | miRNASeq | IlluminaGA_miRNASeq | 3 | bcgsc.ca__IlluminaGA_miRNASeq__TCGA-AA-3986-01A-02T-1021-13__mirna_quantification.txt |
| TCGA-AA-3989-01 | miRNASeq | IlluminaGA_miRNASeq | 3 | bcgsc.ca__IlluminaGA_miRNASeq__TCGA-AA-3989-01A-01T-1021-13__mirna_quantification.txt |
| TCGA-AA-3994-01 | miRNASeq | IlluminaGA_miRNASeq | 3 | bcgsc.ca__IlluminaGA_miRNASeq__TCGA-AA-3994-01A-01T-1114-13__mirna_quantification.txt |
| TCGA-AA-A00L-01 | miRNASeq | IlluminaGA_miRNASeq | 3 | bcgsc.ca__IlluminaGA_miRNASeq__TCGA-AA-A00L-01A-31R-A076-13__mirna_quantification.txt |
| TCGA-AA-A00O-01 | miRNASeq | IlluminaGA_miRNASeq | 3 | bcgsc.ca__IlluminaGA_miRNASeq__TCGA-AA-A00O-01A-11R-A076-13__mirna_quantification.txt |
| TCGA-AA-A00Q-01 | miRNASeq | IlluminaGA_miRNASeq | 3 | bcgsc.ca__IlluminaGA_miRNASeq__TCGA-AA-A00Q-01A-31R-A076-13__mirna_quantification.txt |
| TCGA-AA-A00W-01 | miRNASeq | IlluminaGA_miRNASeq | 3 | bcgsc.ca__IlluminaGA_miRNASeq__TCGA-AA-A00W-01A-21R-A076-13__mirna_quantification.txt |
| TCGA-AA-A010-01 | miRNASeq | IlluminaGA_miRNASeq | 3 | bcgsc.ca__IlluminaGA_miRNASeq__TCGA-AA-A010-01A-54R-A076-13__mirna_quantification.txt |
| TCGA-AA-A01D-01 | miRNASeq | IlluminaGA_miRNASeq | 3 | bcgsc.ca__IlluminaGA_miRNASeq__TCGA-AA-A01D-01A-21R-A076-13__mirna_quantification.txt |
| TCGA-AA-A024-01 | miRNASeq | IlluminaGA_miRNASeq | 3 | bcgsc.ca__IlluminaGA_miRNASeq__TCGA-AA-A024-01A-11R-A076-13__mirna_quantification.txt |
| TCGA-AA-A029-01 | miRNASeq | IlluminaGA_miRNASeq | 3 | bcgsc.ca__IlluminaGA_miRNASeq__TCGA-AA-A029-01A-22R-A076-13__mirna_quantification.txt |
| TCGA-AA-A02E-01 | miRNASeq | IlluminaGA_miRNASeq | 3 | bcgsc.ca__IlluminaGA_miRNASeq__TCGA-AA-A02E-01A-21R-A076-13__mirna_quantification.txt |
| TCGA-AA-A02F-01 | miRNASeq | IlluminaGA_miRNASeq | 3 | bcgsc.ca__IlluminaGA_miRNASeq__TCGA-AA-A02F-01A-21R-A076-13__mirna_quantification.txt |
| TCGA-AA-A02H-01 | miRNASeq | IlluminaGA_miRNASeq | 3 | bcgsc.ca__IlluminaGA_miRNASeq__TCGA-AA-A02H-01A-31R-A076-13__mirna_quantification.txt |
| TCGA-AA-A02J-01 | miRNASeq | IlluminaGA_miRNASeq | 3 | bcgsc.ca__IlluminaGA_miRNASeq__TCGA-AA-A02J-01A-21R-A076-13__mirna_quantification.txt |
| TCGA-AA-A02R-01 | miRNASeq | IlluminaGA_miRNASeq | 3 | bcgsc.ca__IlluminaGA_miRNASeq__TCGA-AA-A02R-01A-21R-A076-13__mirna_quantification.txt |
| TCGA-AY-4070-01 | miRNASeq | IlluminaGA_miRNASeq | 3 | bcgsc.ca__IlluminaGA_miRNASeq__TCGA-AY-4070-01A-01T-1114-13__mirna_quantification.txt |
| TCGA-AY-4071-01 | miRNASeq | IlluminaGA_miRNASeq | 3 | bcgsc.ca__IlluminaGA_miRNASeq__TCGA-AY-4071-01A-01T-1114-13__mirna_quantification.txt |
| TCGA-A6-2670-01 | SNP | Genome_Wide_SNP_6 | 3 | broad.mit.edu__Genome_Wide_SNP_6__TCGA-A6-2670-01A-02D-0819-01__snp_analysis.hg19.seg.txt |
| TCGA-A6-2672-01 | SNP | Genome_Wide_SNP_6 | 3 | broad.mit.edu__Genome_Wide_SNP_6__TCGA-A6-2672-01A-01D-0824-01__snp_analysis.hg19.seg.txt |
| TCGA-A6-2674-01 | SNP | Genome_Wide_SNP_6 | 3 | broad.mit.edu__Genome_Wide_SNP_6__TCGA-A6-2674-01A-02D-0819-01__snp_analysis.hg19.seg.txt |
| TCGA-A6-2676-01 | SNP | Genome_Wide_SNP_6 | 3 | broad.mit.edu__Genome_Wide_SNP_6__TCGA-A6-2676-01A-01D-0824-01__snp_analysis.hg19.seg.txt |
| TCGA-A6-2677-01 | SNP | Genome_Wide_SNP_6 | 3 | broad.mit.edu__Genome_Wide_SNP_6__TCGA-A6-2677-01A-01D-0819-01__snp_analysis.hg19.seg.txt |
| TCGA-A6-2678-01 | SNP | Genome_Wide_SNP_6 | 3 | broad.mit.edu__Genome_Wide_SNP_6__TCGA-A6-2678-01A-01D-0819-01__snp_analysis.hg19.seg.txt |
| TCGA-A6-2683-01 | SNP | Genome_Wide_SNP_6 | 3 | broad.mit.edu__Genome_Wide_SNP_6__TCGA-A6-2683-01A-01D-0819-01__snp_analysis.hg19.seg.txt |
| TCGA-A6-3807-01 | SNP | Genome_Wide_SNP_6 | 3 | broad.mit.edu__Genome_Wide_SNP_6__TCGA-A6-3807-01A-01D-1018-01__snp_analysis.hg19.seg.txt |
| TCGA-A6-3810-01 | SNP | Genome_Wide_SNP_6 | 3 | broad.mit.edu__Genome_Wide_SNP_6__TCGA-A6-3810-01A-01D-1018-01__snp_analysis.hg19.seg.txt |
| TCGA-AA-3514-01 | SNP | Genome_Wide_SNP_6 | 3 | broad.mit.edu__Genome_Wide_SNP_6__TCGA-AA-3514-01A-02D-0819-01__snp_analysis.hg19.seg.txt |
| TCGA-AA-3516-01 | SNP | Genome_Wide_SNP_6 | 3 | broad.mit.edu__Genome_Wide_SNP_6__TCGA-AA-3516-01A-02D-0824-01__snp_analysis.hg19.seg.txt |
| TCGA-AA-3517-01 | SNP | Genome_Wide_SNP_6 | 3 | broad.mit.edu__Genome_Wide_SNP_6__TCGA-AA-3517-01A-01D-0819-01__snp_analysis.hg19.seg.txt |
| TCGA-AA-3518-01 | SNP | Genome_Wide_SNP_6 | 3 | broad.mit.edu__Genome_Wide_SNP_6__TCGA-AA-3518-01A-02D-0824-01__snp_analysis.hg19.seg.txt |
| TCGA-AA-3519-01 | SNP | Genome_Wide_SNP_6 | 3 | broad.mit.edu__Genome_Wide_SNP_6__TCGA-AA-3519-01A-02D-0819-01__snp_analysis.hg19.seg.txt |
| TCGA-AA-3521-01 | SNP | Genome_Wide_SNP_6 | 3 | broad.mit.edu__Genome_Wide_SNP_6__TCGA-AA-3521-01A-01D-0819-01__snp_analysis.hg19.seg.txt |
| TCGA-AA-3522-01 | SNP | Genome_Wide_SNP_6 | 3 | broad.mit.edu__Genome_Wide_SNP_6__TCGA-AA-3522-01A-01D-0819-01__snp_analysis.hg19.seg.txt |
| TCGA-AA-3524-01 | SNP | Genome_Wide_SNP_6 | 3 | broad.mit.edu__Genome_Wide_SNP_6__TCGA-AA-3524-01A-02D-0819-01__snp_analysis.hg19.seg.txt |
| TCGA-AA-3526-01 | SNP | Genome_Wide_SNP_6 | 3 | broad.mit.edu__Genome_Wide_SNP_6__TCGA-AA-3526-01A-02D-0819-01__snp_analysis.hg19.seg.txt |
| TCGA-AA-3527-01 | SNP | Genome_Wide_SNP_6 | 3 | broad.mit.edu__Genome_Wide_SNP_6__TCGA-AA-3527-01A-01D-0819-01__snp_analysis.hg19.seg.txt |
| TCGA-AA-3529-01 | SNP | Genome_Wide_SNP_6 | 3 | broad.mit.edu__Genome_Wide_SNP_6__TCGA-AA-3529-01A-02D-0819-01__snp_analysis.hg19.seg.txt |
| TCGA-AA-3530-01 | SNP | Genome_Wide_SNP_6 | 3 | broad.mit.edu__Genome_Wide_SNP_6__TCGA-AA-3530-01A-01D-1018-01__snp_analysis.hg19.seg.txt |
| TCGA-AA-3531-01 | SNP | Genome_Wide_SNP_6 | 3 | broad.mit.edu__Genome_Wide_SNP_6__TCGA-AA-3531-01A-01D-0819-01__snp_analysis.hg19.seg.txt |
| TCGA-AA-3532-01 | SNP | Genome_Wide_SNP_6 | 3 | broad.mit.edu__Genome_Wide_SNP_6__TCGA-AA-3532-01A-01D-0819-01__snp_analysis.hg19.seg.txt |
| TCGA-AA-3534-01 | SNP | Genome_Wide_SNP_6 | 3 | broad.mit.edu__Genome_Wide_SNP_6__TCGA-AA-3534-01A-01D-0819-01__snp_analysis.hg19.seg.txt |
| TCGA-AA-3538-01 | SNP | Genome_Wide_SNP_6 | 3 | broad.mit.edu__Genome_Wide_SNP_6__TCGA-AA-3538-01A-01D-0819-01__snp_analysis.hg19.seg.txt |
| TCGA-AA-3542-01 | SNP | Genome_Wide_SNP_6 | 3 | broad.mit.edu__Genome_Wide_SNP_6__TCGA-AA-3542-01A-02D-0819-01__snp_analysis.hg19.seg.txt |
| TCGA-AA-3543-01 | SNP | Genome_Wide_SNP_6 | 3 | broad.mit.edu__Genome_Wide_SNP_6__TCGA-AA-3543-01A-01D-0824-01__snp_analysis.hg19.seg.txt |
| TCGA-AA-3544-01 | SNP | Genome_Wide_SNP_6 | 3 | broad.mit.edu__Genome_Wide_SNP_6__TCGA-AA-3544-01A-01D-0819-01__snp_analysis.hg19.seg.txt |
| TCGA-AA-3548-01 | SNP | Genome_Wide_SNP_6 | 3 | broad.mit.edu__Genome_Wide_SNP_6__TCGA-AA-3548-01A-01D-0819-01__snp_analysis.hg19.seg.txt |
| TCGA-AA-3549-01 | SNP | Genome_Wide_SNP_6 | 3 | broad.mit.edu__Genome_Wide_SNP_6__TCGA-AA-3549-01A-02D-0819-01__snp_analysis.hg19.seg.txt |
| TCGA-AA-3552-01 | SNP | Genome_Wide_SNP_6 | 3 | broad.mit.edu__Genome_Wide_SNP_6__TCGA-AA-3552-01A-01D-0819-01__snp_analysis.hg19.seg.txt |
| TCGA-AA-3553-01 | SNP | Genome_Wide_SNP_6 | 3 | broad.mit.edu__Genome_Wide_SNP_6__TCGA-AA-3553-01A-01D-0819-01__snp_analysis.hg19.seg.txt |
| TCGA-AA-3554-01 | SNP | Genome_Wide_SNP_6 | 3 | broad.mit.edu__Genome_Wide_SNP_6__TCGA-AA-3554-01A-01D-0824-01__snp_analysis.hg19.seg.txt |
| TCGA-AA-3555-01 | SNP | Genome_Wide_SNP_6 | 3 | broad.mit.edu__Genome_Wide_SNP_6__TCGA-AA-3555-01A-01D-0819-01__snp_analysis.hg19.seg.txt |
| TCGA-AA-3556-01 | SNP | Genome_Wide_SNP_6 | 3 | broad.mit.edu__Genome_Wide_SNP_6__TCGA-AA-3556-01A-01D-0819-01__snp_analysis.hg19.seg.txt |
| TCGA-AA-3558-01 | SNP | Genome_Wide_SNP_6 | 3 | broad.mit.edu__Genome_Wide_SNP_6__TCGA-AA-3558-01A-01D-0819-01__snp_analysis.hg19.seg.txt |
| TCGA-AA-3560-01 | SNP | Genome_Wide_SNP_6 | 3 | broad.mit.edu__Genome_Wide_SNP_6__TCGA-AA-3560-01A-01D-0819-01__snp_analysis.hg19.seg.txt |
| TCGA-AA-3561-01 | SNP | Genome_Wide_SNP_6 | 3 | broad.mit.edu__Genome_Wide_SNP_6__TCGA-AA-3561-01A-01D-0819-01__snp_analysis.hg19.seg.txt |
| TCGA-AA-3562-01 | SNP | Genome_Wide_SNP_6 | 3 | broad.mit.edu__Genome_Wide_SNP_6__TCGA-AA-3562-01A-02D-0819-01__snp_analysis.hg19.seg.txt |
| TCGA-AA-3664-01 | SNP | Genome_Wide_SNP_6 | 3 | broad.mit.edu__Genome_Wide_SNP_6__TCGA-AA-3664-01A-01D-0903-01__snp_analysis.hg19.seg.txt |
| TCGA-AA-3666-01 | SNP | Genome_Wide_SNP_6 | 3 | broad.mit.edu__Genome_Wide_SNP_6__TCGA-AA-3666-01A-02D-0903-01__snp_analysis.hg19.seg.txt |
| TCGA-AA-3667-01 | SNP | Genome_Wide_SNP_6 | 3 | broad.mit.edu__Genome_Wide_SNP_6__TCGA-AA-3667-01A-01D-0903-01__snp_analysis.hg19.seg.txt |
| TCGA-AA-3672-01 | SNP | Genome_Wide_SNP_6 | 3 | broad.mit.edu__Genome_Wide_SNP_6__TCGA-AA-3672-01A-01D-0903-01__snp_analysis.hg19.seg.txt |
| TCGA-AA-3673-01 | SNP | Genome_Wide_SNP_6 | 3 | broad.mit.edu__Genome_Wide_SNP_6__TCGA-AA-3673-01A-01D-0903-01__snp_analysis.hg19.seg.txt |
| TCGA-AA-3678-01 | SNP | Genome_Wide_SNP_6 | 3 | broad.mit.edu__Genome_Wide_SNP_6__TCGA-AA-3678-01A-01D-0903-01__snp_analysis.hg19.seg.txt |
| TCGA-AA-3679-01 | SNP | Genome_Wide_SNP_6 | 3 | broad.mit.edu__Genome_Wide_SNP_6__TCGA-AA-3679-01A-02D-0903-01__snp_analysis.hg19.seg.txt |
| TCGA-AA-3680-01 | SNP | Genome_Wide_SNP_6 | 3 | broad.mit.edu__Genome_Wide_SNP_6__TCGA-AA-3680-01A-01D-0903-01__snp_analysis.hg19.seg.txt |
| TCGA-AA-3681-01 | SNP | Genome_Wide_SNP_6 | 3 | broad.mit.edu__Genome_Wide_SNP_6__TCGA-AA-3681-01A-01D-0903-01__snp_analysis.hg19.seg.txt |
| TCGA-AA-3684-01 | SNP | Genome_Wide_SNP_6 | 3 | broad.mit.edu__Genome_Wide_SNP_6__TCGA-AA-3684-01A-02D-0903-01__snp_analysis.hg19.seg.txt |
| TCGA-AA-3685-01 | SNP | Genome_Wide_SNP_6 | 3 | broad.mit.edu__Genome_Wide_SNP_6__TCGA-AA-3685-01A-02D-0903-01__snp_analysis.hg19.seg.txt |
| TCGA-AA-3688-01 | SNP | Genome_Wide_SNP_6 | 3 | broad.mit.edu__Genome_Wide_SNP_6__TCGA-AA-3688-01A-01D-0903-01__snp_analysis.hg19.seg.txt |
| TCGA-AA-3692-01 | SNP | Genome_Wide_SNP_6 | 3 | broad.mit.edu__Genome_Wide_SNP_6__TCGA-AA-3692-01A-01D-0903-01__snp_analysis.hg19.seg.txt |
| TCGA-AA-3693-01 | SNP | Genome_Wide_SNP_6 | 3 | broad.mit.edu__Genome_Wide_SNP_6__TCGA-AA-3693-01A-01D-0903-01__snp_analysis.hg19.seg.txt |
| TCGA-AA-3695-01 | SNP | Genome_Wide_SNP_6 | 3 | broad.mit.edu__Genome_Wide_SNP_6__TCGA-AA-3695-01A-01D-0903-01__snp_analysis.hg19.seg.txt |
| TCGA-AA-3696-01 | SNP | Genome_Wide_SNP_6 | 3 | broad.mit.edu__Genome_Wide_SNP_6__TCGA-AA-3696-01A-01D-0903-01__snp_analysis.hg19.seg.txt |
| TCGA-AA-3710-01 | SNP | Genome_Wide_SNP_6 | 3 | broad.mit.edu__Genome_Wide_SNP_6__TCGA-AA-3710-01A-01D-1018-01__snp_analysis.hg19.seg.txt |
| TCGA-AA-3715-01 | SNP | Genome_Wide_SNP_6 | 3 | broad.mit.edu__Genome_Wide_SNP_6__TCGA-AA-3715-01A-01D-0903-01__snp_analysis.hg19.seg.txt |
| TCGA-AA-3811-01 | SNP | Genome_Wide_SNP_6 | 3 | broad.mit.edu__Genome_Wide_SNP_6__TCGA-AA-3811-01A-01D-1018-01__snp_analysis.hg19.seg.txt |
| TCGA-AA-3812-01 | SNP | Genome_Wide_SNP_6 | 3 | broad.mit.edu__Genome_Wide_SNP_6__TCGA-AA-3812-01A-01D-0903-01__snp_analysis.hg19.seg.txt |
| TCGA-AA-3814-01 | SNP | Genome_Wide_SNP_6 | 3 | broad.mit.edu__Genome_Wide_SNP_6__TCGA-AA-3814-01A-01D-0903-01__snp_analysis.hg19.seg.txt |
| TCGA-AA-3818-01 | SNP | Genome_Wide_SNP_6 | 3 | broad.mit.edu__Genome_Wide_SNP_6__TCGA-AA-3818-01A-01D-0903-01__snp_analysis.hg19.seg.txt |
| TCGA-AA-3819-01 | SNP | Genome_Wide_SNP_6 | 3 | broad.mit.edu__Genome_Wide_SNP_6__TCGA-AA-3819-01A-01D-0903-01__snp_analysis.hg19.seg.txt |
| TCGA-AA-3821-01 | SNP | Genome_Wide_SNP_6 | 3 | broad.mit.edu__Genome_Wide_SNP_6__TCGA-AA-3821-01A-01D-1018-01__snp_analysis.hg19.seg.txt |
| TCGA-AA-3831-01 | SNP | Genome_Wide_SNP_6 | 3 | broad.mit.edu__Genome_Wide_SNP_6__TCGA-AA-3831-01A-01D-0903-01__snp_analysis.hg19.seg.txt |
| TCGA-AA-3833-01 | SNP | Genome_Wide_SNP_6 | 3 | broad.mit.edu__Genome_Wide_SNP_6__TCGA-AA-3833-01A-01D-0903-01__snp_analysis.hg19.seg.txt |
| TCGA-AA-3837-01 | SNP | Genome_Wide_SNP_6 | 3 | broad.mit.edu__Genome_Wide_SNP_6__TCGA-AA-3837-01A-01D-0903-01__snp_analysis.hg19.seg.txt |
| TCGA-AA-3842-01 | SNP | Genome_Wide_SNP_6 | 3 | broad.mit.edu__Genome_Wide_SNP_6__TCGA-AA-3842-01A-01D-1018-01__snp_analysis.hg19.seg.txt |
| TCGA-AA-3844-01 | SNP | Genome_Wide_SNP_6 | 3 | broad.mit.edu__Genome_Wide_SNP_6__TCGA-AA-3844-01A-01D-1018-01__snp_analysis.hg19.seg.txt |
| TCGA-AA-3845-01 | SNP | Genome_Wide_SNP_6 | 3 | broad.mit.edu__Genome_Wide_SNP_6__TCGA-AA-3845-01A-01D-1018-01__snp_analysis.hg19.seg.txt |
| TCGA-AA-3846-01 | SNP | Genome_Wide_SNP_6 | 3 | broad.mit.edu__Genome_Wide_SNP_6__TCGA-AA-3846-01A-01D-1018-01__snp_analysis.hg19.seg.txt |
| TCGA-AA-3848-01 | SNP | Genome_Wide_SNP_6 | 3 | broad.mit.edu__Genome_Wide_SNP_6__TCGA-AA-3848-01A-01D-0903-01__snp_analysis.hg19.seg.txt |
| TCGA-AA-3850-01 | SNP | Genome_Wide_SNP_6 | 3 | broad.mit.edu__Genome_Wide_SNP_6__TCGA-AA-3850-01A-01D-1018-01__snp_analysis.hg19.seg.txt |
| TCGA-AA-3851-01 | SNP | Genome_Wide_SNP_6 | 3 | broad.mit.edu__Genome_Wide_SNP_6__TCGA-AA-3851-01A-01D-1018-01__snp_analysis.hg19.seg.txt |
| TCGA-AA-3852-01 | SNP | Genome_Wide_SNP_6 | 3 | broad.mit.edu__Genome_Wide_SNP_6__TCGA-AA-3852-01A-01D-0903-01__snp_analysis.hg19.seg.txt |
| TCGA-AA-3854-01 | SNP | Genome_Wide_SNP_6 | 3 | broad.mit.edu__Genome_Wide_SNP_6__TCGA-AA-3854-01A-01D-0903-01__snp_analysis.hg19.seg.txt |
| TCGA-AA-3855-01 | SNP | Genome_Wide_SNP_6 | 3 | broad.mit.edu__Genome_Wide_SNP_6__TCGA-AA-3855-01A-01D-1018-01__snp_analysis.hg19.seg.txt |
| TCGA-AA-3856-01 | SNP | Genome_Wide_SNP_6 | 3 | broad.mit.edu__Genome_Wide_SNP_6__TCGA-AA-3856-01A-01D-0903-01__snp_analysis.hg19.seg.txt |
| TCGA-AA-3858-01 | SNP | Genome_Wide_SNP_6 | 3 | broad.mit.edu__Genome_Wide_SNP_6__TCGA-AA-3858-01A-01D-0903-01__snp_analysis.hg19.seg.txt |
| TCGA-AA-3860-01 | SNP | Genome_Wide_SNP_6 | 3 | broad.mit.edu__Genome_Wide_SNP_6__TCGA-AA-3860-01A-02D-0903-01__snp_analysis.hg19.seg.txt |
| TCGA-AA-3864-01 | SNP | Genome_Wide_SNP_6 | 3 | broad.mit.edu__Genome_Wide_SNP_6__TCGA-AA-3864-01A-01D-1018-01__snp_analysis.hg19.seg.txt |
| TCGA-AA-3866-01 | SNP | Genome_Wide_SNP_6 | 3 | broad.mit.edu__Genome_Wide_SNP_6__TCGA-AA-3866-01A-01D-1018-01__snp_analysis.hg19.seg.txt |
| TCGA-AA-3867-01 | SNP | Genome_Wide_SNP_6 | 3 | broad.mit.edu__Genome_Wide_SNP_6__TCGA-AA-3867-01A-01D-1018-01__snp_analysis.hg19.seg.txt |
| TCGA-AA-3869-01 | SNP | Genome_Wide_SNP_6 | 3 | broad.mit.edu__Genome_Wide_SNP_6__TCGA-AA-3869-01A-01D-1018-01__snp_analysis.hg19.seg.txt |
| TCGA-AA-3870-01 | SNP | Genome_Wide_SNP_6 | 3 | broad.mit.edu__Genome_Wide_SNP_6__TCGA-AA-3870-01A-01D-1018-01__snp_analysis.hg19.seg.txt |
| TCGA-AA-3872-01 | SNP | Genome_Wide_SNP_6 | 3 | broad.mit.edu__Genome_Wide_SNP_6__TCGA-AA-3872-01A-01D-1018-01__snp_analysis.hg19.seg.txt |
| TCGA-AA-3875-01 | SNP | Genome_Wide_SNP_6 | 3 | broad.mit.edu__Genome_Wide_SNP_6__TCGA-AA-3875-01A-01D-0903-01__snp_analysis.hg19.seg.txt |
| TCGA-AA-3877-01 | SNP | Genome_Wide_SNP_6 | 3 | broad.mit.edu__Genome_Wide_SNP_6__TCGA-AA-3877-01A-01D-1018-01__snp_analysis.hg19.seg.txt |
| TCGA-AA-3930-01 | SNP | Genome_Wide_SNP_6 | 3 | broad.mit.edu__Genome_Wide_SNP_6__TCGA-AA-3930-01A-01D-1018-01__snp_analysis.hg19.seg.txt |
| TCGA-AA-3939-01 | SNP | Genome_Wide_SNP_6 | 3 | broad.mit.edu__Genome_Wide_SNP_6__TCGA-AA-3939-01A-01D-1018-01__snp_analysis.hg19.seg.txt |
| TCGA-AA-3941-01 | SNP | Genome_Wide_SNP_6 | 3 | broad.mit.edu__Genome_Wide_SNP_6__TCGA-AA-3941-01A-01D-1018-01__snp_analysis.hg19.seg.txt |
| TCGA-AA-3947-01 | SNP | Genome_Wide_SNP_6 | 3 | broad.mit.edu__Genome_Wide_SNP_6__TCGA-AA-3947-01A-01D-1018-01__snp_analysis.hg19.seg.txt |
| TCGA-AA-3949-01 | SNP | Genome_Wide_SNP_6 | 3 | broad.mit.edu__Genome_Wide_SNP_6__TCGA-AA-3949-01A-01D-1018-01__snp_analysis.hg19.seg.txt |
| TCGA-AA-3950-01 | SNP | Genome_Wide_SNP_6 | 3 | broad.mit.edu__Genome_Wide_SNP_6__TCGA-AA-3950-01A-02D-1018-01__snp_analysis.hg19.seg.txt |
| TCGA-AA-3952-01 | SNP | Genome_Wide_SNP_6 | 3 | broad.mit.edu__Genome_Wide_SNP_6__TCGA-AA-3952-01A-01D-1018-01__snp_analysis.hg19.seg.txt |
| TCGA-AA-3955-01 | SNP | Genome_Wide_SNP_6 | 3 | broad.mit.edu__Genome_Wide_SNP_6__TCGA-AA-3955-01A-02D-1018-01__snp_analysis.hg19.seg.txt |
| TCGA-AA-3956-01 | SNP | Genome_Wide_SNP_6 | 3 | broad.mit.edu__Genome_Wide_SNP_6__TCGA-AA-3956-01A-02D-1018-01__snp_analysis.hg19.seg.txt |
| TCGA-AA-3966-01 | SNP | Genome_Wide_SNP_6 | 3 | broad.mit.edu__Genome_Wide_SNP_6__TCGA-AA-3966-01A-01D-1427-01__snp_analysis.hg19.seg.txt |
| TCGA-AA-3968-01 | SNP | Genome_Wide_SNP_6 | 3 | broad.mit.edu__Genome_Wide_SNP_6__TCGA-AA-3968-01A-01D-1018-01__snp_analysis.hg19.seg.txt |
| TCGA-AA-3971-01 | SNP | Genome_Wide_SNP_6 | 3 | broad.mit.edu__Genome_Wide_SNP_6__TCGA-AA-3971-01A-01D-1018-01__snp_analysis.hg19.seg.txt |
| TCGA-AA-3972-01 | SNP | Genome_Wide_SNP_6 | 3 | broad.mit.edu__Genome_Wide_SNP_6__TCGA-AA-3972-01A-01D-1018-01__snp_analysis.hg19.seg.txt |
| TCGA-AA-3973-01 | SNP | Genome_Wide_SNP_6 | 3 | broad.mit.edu__Genome_Wide_SNP_6__TCGA-AA-3973-01A-01D-1018-01__snp_analysis.hg19.seg.txt |
| TCGA-AA-3975-01 | SNP | Genome_Wide_SNP_6 | 3 | broad.mit.edu__Genome_Wide_SNP_6__TCGA-AA-3975-01A-01D-1018-01__snp_analysis.hg19.seg.txt |
| TCGA-AA-3976-01 | SNP | Genome_Wide_SNP_6 | 3 | broad.mit.edu__Genome_Wide_SNP_6__TCGA-AA-3976-01A-01D-1018-01__snp_analysis.hg19.seg.txt |
| TCGA-AA-3977-01 | SNP | Genome_Wide_SNP_6 | 3 | broad.mit.edu__Genome_Wide_SNP_6__TCGA-AA-3977-01A-01D-1018-01__snp_analysis.hg19.seg.txt |
| TCGA-AA-3979-01 | SNP | Genome_Wide_SNP_6 | 3 | broad.mit.edu__Genome_Wide_SNP_6__TCGA-AA-3979-01A-01D-1018-01__snp_analysis.hg19.seg.txt |
| TCGA-AA-3982-01 | SNP | Genome_Wide_SNP_6 | 3 | broad.mit.edu__Genome_Wide_SNP_6__TCGA-AA-3982-01A-02D-1018-01__snp_analysis.hg19.seg.txt |
| TCGA-AA-3984-01 | SNP | Genome_Wide_SNP_6 | 3 | broad.mit.edu__Genome_Wide_SNP_6__TCGA-AA-3984-01A-02D-1018-01__snp_analysis.hg19.seg.txt |
| TCGA-AA-3986-01 | SNP | Genome_Wide_SNP_6 | 3 | broad.mit.edu__Genome_Wide_SNP_6__TCGA-AA-3986-01A-02D-1018-01__snp_analysis.hg19.seg.txt |
| TCGA-AA-3989-01 | SNP | Genome_Wide_SNP_6 | 3 | broad.mit.edu__Genome_Wide_SNP_6__TCGA-AA-3989-01A-01D-1019-01__snp_analysis.hg19.seg.txt |
| TCGA-AA-3994-01 | SNP | Genome_Wide_SNP_6 | 3 | broad.mit.edu__Genome_Wide_SNP_6__TCGA-AA-3994-01A-01D-1427-01__snp_analysis.hg19.seg.txt |
| TCGA-AA-A00L-01 | SNP | Genome_Wide_SNP_6 | 3 | broad.mit.edu__Genome_Wide_SNP_6__TCGA-AA-A00L-01A-01D-A003-01__snp_analysis.hg19.seg.txt |
| TCGA-AA-A00O-01 | SNP | Genome_Wide_SNP_6 | 3 | broad.mit.edu__Genome_Wide_SNP_6__TCGA-AA-A00O-01A-02D-A008-01__snp_analysis.hg19.seg.txt |
| TCGA-AA-A00Q-01 | SNP | Genome_Wide_SNP_6 | 3 | broad.mit.edu__Genome_Wide_SNP_6__TCGA-AA-A00Q-01A-01D-A003-01__snp_analysis.hg19.seg.txt |
| TCGA-AA-A00W-01 | SNP | Genome_Wide_SNP_6 | 3 | broad.mit.edu__Genome_Wide_SNP_6__TCGA-AA-A00W-01A-01D-A003-01__snp_analysis.hg19.seg.txt |
| TCGA-AA-A010-01 | SNP | Genome_Wide_SNP_6 | 3 | broad.mit.edu__Genome_Wide_SNP_6__TCGA-AA-A010-01A-01D-A008-01__snp_analysis.hg19.seg.txt |
| TCGA-AA-A01D-01 | SNP | Genome_Wide_SNP_6 | 3 | broad.mit.edu__Genome_Wide_SNP_6__TCGA-AA-A01D-01A-01D-A008-01__snp_analysis.hg19.seg.txt |
| TCGA-AA-A024-01 | SNP | Genome_Wide_SNP_6 | 3 | broad.mit.edu__Genome_Wide_SNP_6__TCGA-AA-A024-01A-02D-A008-01__snp_analysis.hg19.seg.txt |
| TCGA-AA-A029-01 | SNP | Genome_Wide_SNP_6 | 3 | broad.mit.edu__Genome_Wide_SNP_6__TCGA-AA-A029-01A-01D-A008-01__snp_analysis.hg19.seg.txt |
| TCGA-AA-A02E-01 | SNP | Genome_Wide_SNP_6 | 3 | broad.mit.edu__Genome_Wide_SNP_6__TCGA-AA-A02E-01A-01D-A008-01__snp_analysis.hg19.seg.txt |
| TCGA-AA-A02F-01 | SNP | Genome_Wide_SNP_6 | 3 | broad.mit.edu__Genome_Wide_SNP_6__TCGA-AA-A02F-01A-01D-A008-01__snp_analysis.hg19.seg.txt |
| TCGA-AA-A02H-01 | SNP | Genome_Wide_SNP_6 | 3 | broad.mit.edu__Genome_Wide_SNP_6__TCGA-AA-A02H-01A-01D-A008-01__snp_analysis.hg19.seg.txt |
| TCGA-AA-A02J-01 | SNP | Genome_Wide_SNP_6 | 3 | broad.mit.edu__Genome_Wide_SNP_6__TCGA-AA-A02J-01A-01D-A008-01__snp_analysis.hg19.seg.txt |
| TCGA-AA-A02R-01 | SNP | Genome_Wide_SNP_6 | 3 | broad.mit.edu__Genome_Wide_SNP_6__TCGA-AA-A02R-01A-01D-A008-01__snp_analysis.hg19.seg.txt |
| TCGA-AY-4070-01 | SNP | Genome_Wide_SNP_6 | 3 | broad.mit.edu__Genome_Wide_SNP_6__TCGA-AY-4070-01A-01D-1427-01__snp_analysis.hg19.seg.txt |
| TCGA-AY-4071-01 | SNP | Genome_Wide_SNP_6 | 3 | broad.mit.edu__Genome_Wide_SNP_6__TCGA-AY-4071-01A-01D-1427-01__snp_analysis.hg19.seg.txt |
